# Interventions for vector-borne diseases focused on housing and hygiene in urban areas: a scoping review

**DOI:** 10.1186/s40249-018-0477-5

**Published:** 2018-09-03

**Authors:** Stéphanie Degroote, Kate Zinszer, Valéry Ridde

**Affiliations:** 1 University of Montreal Public Health Research Institute, Montreal, Canada; 2 French Institute For Research on sustainable Development (IRD), IRD Paris Descartes University (CEPED), Paris Sorbonne Cities University, Erl Inserm Sagesud, Paris, France

**Keywords:** Vector-borne disease, Urban area, Housing, Hygiene, Sanitation, Waste management, Prevention, Systematic mixed method review

## Abstract

**Background:**

Over half the world’s human populations are currently at risk from vector-borne diseases (VBDs), and the heaviest burden is borne by the world’s poorest people, communities, and countries. The aim of this study was to conduct a review on VBD interventions relevant to housing and hygiene (including sanitation and waste management) in urban areas.

**Main body:**

We conducted a scoping review, which involved systematically searching peer-reviewed and grey literature published between 2000 and 2016 using five scientific databases and one database for grey literature. Different data extraction tools were used for data coding and extraction. We assessed the quality of each study using the Mixed Methods Appraisal Tool and extracted descriptive characteristics and data about implementation process and transferability from all studies using the Template for Intervention Description and Replication and ASTAIRE (a tool for analyzing the transferability of health promotion interventions) tools.

We reviewed 44 studies. Overall, the studies were judged to be of high risk for bias. Our results suggest multifaceted interventions, particularly community-based interventions, have the potential to achieve wider and more sustained effects than do standard vertical single-component programs. The evaluations of multifaceted interventions tend to include integrated evaluations, using not only entomological indicators but also acceptability and sustainability indicators.

**Conclusions:**

This review highlighted the important need for higher quality research in VBDs and improved and standardized reporting of interventions. Significant research gaps were found regarding qualitative research and implementation research, and results highlighted the need for more interventions focus on sanitation and hygiene practices.

**Electronic supplementary material:**

The online version of this article (10.1186/s40249-018-0477-5) contains supplementary material, which is available to authorized users.

## Multilingual abstracts

Please see Additional file 1 for translation of the abstract into the five official working languages of the United Nations.

## Background

Over half the world’s human populations are currently at risk from vector-borne diseases (VBDs), and the heaviest burden is borne by the world’s poorest people, communities, and countries [1]. Thus, VBDs are disproportionately high in low- and middle-income countries (LMICs) in tropical and subtropical regions, where medical resources for the population are often limited [2]. These diseases also exacerbate poverty, given that illness and disability prevent people from working and supporting themselves and their family, causing further hardship and impeding economic development [3, 4]. The prevention and control of VBDs is not only a health matter but is also essential to improve the socio-economic conditions of LMICs.

The discovery and massive use of residual insecticides targeted at mosquito vectors began in the 1940s and greatly contributed to the success of early vector control campaigns in the Americas, Pacific islands, and Asia [5]. Over several decades, certain VBDs were effectively controlled, and by the 1960s, VBDs were no longer considered significant public health problems outside of Africa. Unfortunately, the benefits of such programs were short-lived, and during the 1970s *Aedes aegypti* (the vector for dengue, chikungunya, and Zika viruses) re-infested most of the countries where it had been previously eliminated [6]. This led to a transition in public health strategy that was initially focused on eradication to one of control. In the absence of vaccines and prophylaxis options, a vector control strategy is the only preventive strategy for VBDs at the moment, with the exception of malaria and dengue vaccines that are being used in small scale contexts. [1]. Unfortunately, we continue to experience an expansion of vector populations, which are becoming increasingly resistant to insecticides [7]. Despite failures of past attempts at vector eradication campaigns and important indications of resistance, mass spraying and larvicides remain the principal control method used in routine practice and in outbreak situations [8]. There is a critical need for alternative preventive measures that are effective and sustainable for VBDs.

Multiple factors influence the geographical dispersion of VBDs, such as environmental changes and globalization, with perhaps the most important drivers being the global population explosion associated with unplanned urbanization [9]. The United Nations Department of Economic and Social Affairs (UNDESA) reports that 54% of the world’s population lives in urban areas and is projected to reach 66% by 2050 [10]. LMICs will continue to experience an unprecedented pace of urbanization with unplanned urban growth, posing significant challenges for human health and sustainable development [11]. Rapid urban growth is dramatically surpassing the capacity of most cities in LMICs to provide adequate water and sanitation services for their citizens [12]. Progress has been made since 1990, with the number of people gaining access to improved sanitation rising from 54% to 68% globally [13], although there remain important inequities in access along the sociodemographic spectrum [13, 14]. Consequently, in rapidly growing urban slums, VBDs and other neglected tropical diseases are thriving [3]. Urban slums are characterized by high population density, absence of urban planning, unsustainable housing, inadequate infrastructure for water and sanitation, and poverty. The proliferation of water containers, which are used to cope with disruptions in piped water access or to collect rainwater, and also discarded items such as used tires, provide plentiful breeding sites for mosquitoes in urban slums, increasing the risk of transmission of several VBDs.

The objective of the present study was to conduct a scoping review to synthesize existing evidence on VBD interventions in urban environments related to housing, hygiene, sanitation, and waste management measures. The purpose was to identify the extent of the literature and to determine the research gaps and priorities for future research.

## Methods

### Research topic

This study is part of a larger series of six scoping reviews conducted by the the “VEctor boRne DiseAses Scoping reviews” (VERDAS) consortium. The protocol of the VERDAS consortium is published [15] but briefly we used an eDelphi survey to select the six topics considered of highest priority by a panel of 84 international experts (43% researchers; 52% public health decision-makers; 5% from the private sector). The eDelphi was a three-round process: 1) panelists suggested topics to be considered; 2) panelists then rated the more than 80 suggested topics from “1–eliminate” to “5–top priority”; and 3) the 20 subjects rated 4 or 5 by more than 65% of the participants were rated a second time. By the end of the third round, the present topic had obtained the mean rate of 3.88 ± 1.07 and ranked the sixth (63.27% of panelists rated it 4 or 5).

### Search strategy

Our search strategy was validated by a public health librarian at the University of Montreal. We conducted a systematic literature search using four scientific electronic databases (PubMed, Embase, Global Health, and the Cochrane Database of Systematic Reviews) and one grey literature database (WHO library database). Finally, we searched reference lists of included articles to find additional relevant articles. Our search strategy consisted of the following combinations of key concepts “Vector-borne diseases” AND “Urban area” AND “prevention and control” AND [“housing” OR “hygiene” OR “sanitation” OR “waste management”]. We included all possible associated keywords to each key concept and appropriate descriptors for each database (see complete search strategy in Additional file 2).

### Selection of relevant studies

In a pilot round of screening, three reviewers (SD, NK, DD) independently screened and evaluated the relevance of the titles and abstracts of 20 references. This enabled the development of post hoc eligibility criteria and ensured consistency between the two reviewers (NK, DD) in the selection of studies. These criteria were consistently applied during the full process of screening. After the independent title and abstract screening by two reviewers (NK, DD), the full texts of the included articles were screened by the same two reviewers. A third reviewer (SD) resolved any discrepancies at each stage of the selection process.

The inclusion criteria were: 1) presents an intervention within a routine context, as opposed to an intervention in response to an outbreak; 2) presents an intervention focused on housing and/or on hygiene (including sanitation and waste management); 3) based in an urban context; 4) published between January 2000 and July 2016; and 5) language of publication: English, French, or Spanish.

Articles were excluded if they: 1) included only epidemiological or prevalence data without a link to a specific intervention; 2) included only entomological surveillance without a link to a specific intervention; 3) used an experimental design to evaluate effectiveness of potential/new vector control measures (dose-effectiveness studies); or 4) were not available in full text versions.

Items that were not original research (e.g. reviews, comments, editorials) were excluded, but references lists were checked for potential relevant original studies.

#### Operational definitions

We defined key concepts to assist with applying the selection criteria. ‘Vector-borne diseases’ (VBDs) are illnesses caused by vectors such as mosquitoes, ticks, and lice that transmit infective pathogens (bacteria, virus, and fungus) from one host (human, birds and animals) to another [3]. We based our list of VBDs on the list provided by the World Health Organization (WHO) [16]. To select interventions specifically within an urban context, we used data from the 2014 World Urbanization Prospects issued by the Population Division of the UNDESA to determine urban populations according to criteria set by each specific country [17].

We adopted the operational definition of ‘infection prevention and control’ from WHO: “Infection prevention and control measures aim to ensure the protection of those who might be vulnerable to acquiring an infection both in the general community and while receiving care” [18]. In accordance with this definition, we focused on interventions occurring within a routine context rather than in a massive and/or emergency response to an outbreak. We therefore included studies that contained interventions focused solely on the reduction of vector populations, even if no specific epidemiological data were provided, as long as the intervention was population-based and not in experimental conditions. Moreover, we focused on interventions relevant to one or the other of two key concepts: 1) *housing*: defined as an intervention taking place in a housing unit, defined as “a place…intended for habitation by a single household, or one not intended for habitation but occupied as living quarters by a household” [19]; 2) *hygiene*: defined by WHO as “practices that help to maintain health and prevent the spread of diseases”, including environmental cleaning, personal hygiene, and sanitation [20]. The term ‘sanitation’ refers to the maintenance of hygienic conditions, through services and actions required for proper handling of waste materials, such as garbage collection and wastewater disposal [21].

### Data extraction, charting and summarizing the findings

We used a standardized Excel (Version 2016, Microsoft Corporation, Richmond, WA, USA) spreadsheet template across our consortium to extract information from included studies. The data extraction consisted of five sections: 1) descriptive characteristics of the included studies; 2) methodological quality assessment using the Mixed Methods Appraisal Tool (MMAT) [22]; 3) macro data extraction using the Template for Intervention Description and Replication (TIDieR) tool [23]; 4) micro data extraction using the ASTAIRE tool (a tool for analyzing the transferability of health promotion interventions) [24]; and 5) additional columns such as ‘challenge faced’ and ‘recommendations’.

## Results

### Search findings

**Fig. 1:**
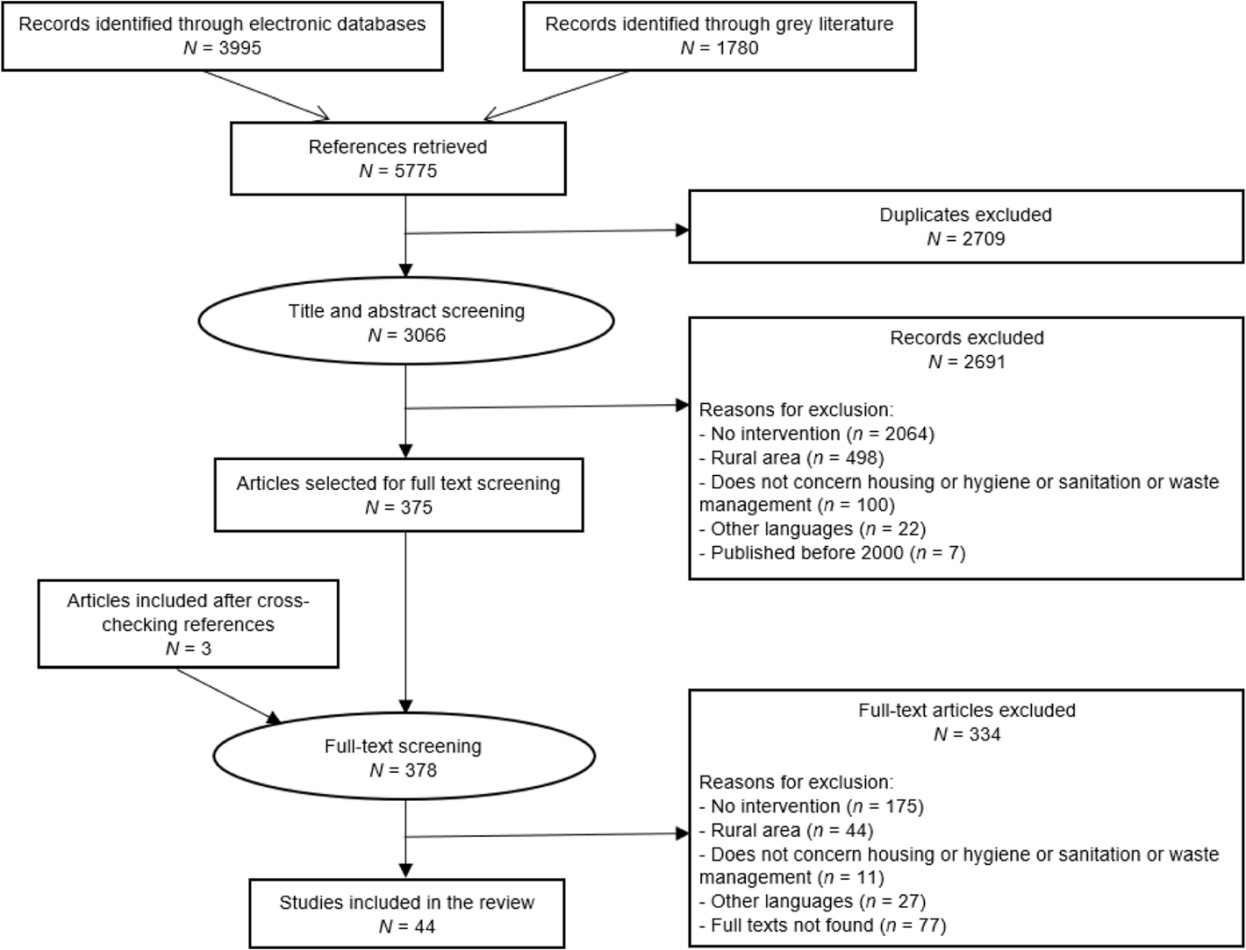
Prisma flow chart of selection process of the included and excluded studies

Our search strategy yielded a total of 5775 citations (3995 from five electronic databases and 1780 from grey literature). That number was reduced to 3066 after excluding 2709 duplicate records. After screening the abstracts of all 3066 citations, we retained 378 articles for full-text screening. A final set of 44 articles met all inclusion criteria and were included in our review. Figure 1 presents the Prisma chart of our study selection process.

### Descriptive characteristics of the studies

The descriptive characteristics are presented in Table 1, where the included studies are classified as either single-component (*n =* 24; 55%) or multi-component interventions (*n =* 20; 45%). We defined the former as interventions based upon only one activity and the latter as referring to a set of simultaneous or sequential activities. This classification was inductive and decided upon after the data extraction to guide the presentation of results and to highlight key differences in complex interventions.

**Table 1:** Descriptive characteristics of interventions

	Total	Single-component (*n =* 24)	Multi-component (*n =* 20)
VBD specific	17	9	9
Dengue	11	4	7
Malaria	3	2	1
Leishmaniasis	2	1	1
Plague	1	1	0
Not VBD specific	27	15	11
*Ae. aegypti* (for dengue and/or chikungunya)	20	10	10
Other (*Ae. aldopictus*, *Culex pipiens*, not specified)	7	6	1
Vectors			
Mosquitoes	41	23	19
Sandflies	2	1	1
Fleas	1	1	
Study design			
Quantitative RCT	13	7	6
Quantitative non-randomized/observational	12	10	2
Quantitative descriptive	11	7	4
Mixed-methods	7	1	6
Qualitative	1	0	1
Types of evaluation indicators			
Entomological indicators	42	22	20
Serological or epidemiological indicators	11	4	7
Population-based indicators (acceptability, use, knowledge, behaviour change, etc.)	21	6	15
Cost information	8	3	5
Integrative nature of evaluations			
Only 1 type of indicator used in evaluation	23	18	4
2 or more types of indicators used in evaluation	21	6	16

**Fig. 2:**
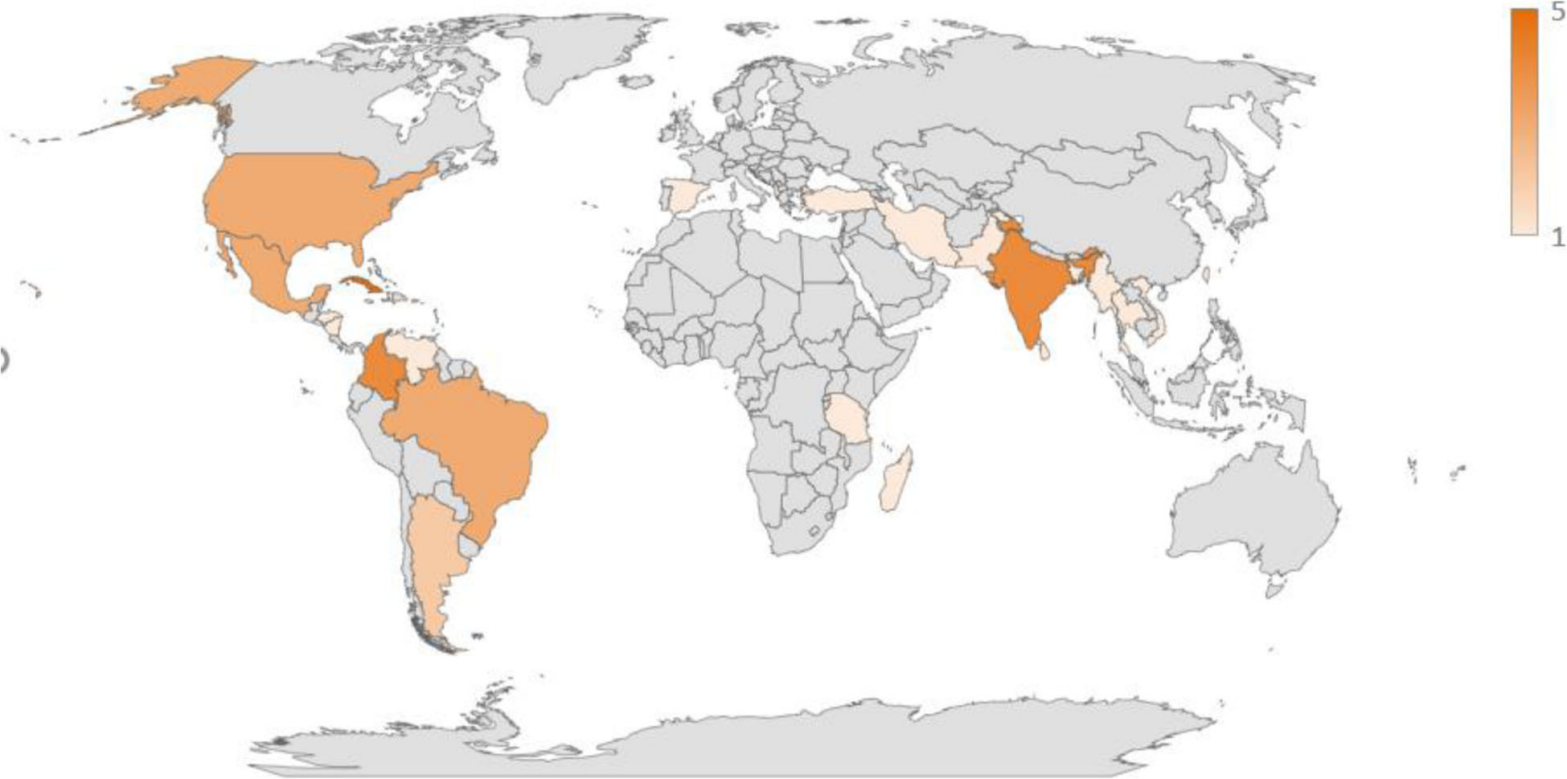
Choropleth map of the geographic distribution of included studies in the scoping review. From 1 study included by country (very light orange) to 5 studies included by country (dark orange)

Of the 44 studies, 38 were published in English (87%), five in Spanish (11%) and one in French (2%). The geographic zones predominantly under study were Latin and Central America (*n =* 12; 27%), the Caribbean (*n =* 9; 21%), and Asia (*n =* 10; 22%), followed by North America (*n =* 6; 13%), the Middle East (*n =* 3; 7%), Africa (*n =* 2; 4%), Oceania (*n =* 2; 4%) and Europe (*n =* 1; 2%) (Fig. 2). Almost all studies were focused on mosquito vector populations (*n =* 41; 93%); only three studies were based on other vector populations: two on sandflies and one on fleas (and, by proxy, rats).

Fewer than half of the studies (*n =* 17; 39%) were focused on one VBD, with dengue being the predominant focus (*n =* 11; or 65% of VBD-specific studies) [6, 25–34], followed by malaria (*n =* 3; 17%) [35–37], leishmaniasis (*n =* 2; 11%) [38, 39], and plague (*n =* 1; 5%) [40]. More than half of the studies (*n =* 27; 61%) did not address a specific VBD and instead used indicators only from the vector population. The majority of non-VBD-specific studies were focused on *Ae. aegypti* (*n =* 20; 74%) [41–60], which is a primary vector for dengue, chikungunya, and Zika transmission. Therefore, there were a total of 31 papers (70%) focused specifically on VBDs transmitted by *Ae. aegypti*.

There was heterogeneity in study designs (based on the MMAT classification), which included 13 quantitative randomized controlled trials (RCT) (30%) [28, 30, 38–40, 42, 47, 48, 53, 55, 59–61], 12 quantitative non-randomized controlled trials or observational studies (27%) [25, 31, 35, 37, 41, 43, 44, 46, 62–65], 11 quantitative descriptive studies (25%) with no control group, using a pre/post design approach [6, 26, 27, 32, 45, 49, 50, 54, 56, 66, 67], seven mixed-methods studies (quantitative and qualitative data) (16%)—among which five were cluster randomized controlled trials [29, 51, 52, 57, 58], one a non-randomized controlled trial [34] and one a descriptive study [36]—and, finally, one qualitative study [33]. Of note, almost all mixed-methods studies were multi-component studies [29, 34, 51, 52, 57, 58], and only one was single-component [36]; also, mixed-methods was the most frequent design for multi-component interventions (*n =* 6; 30%), with the same number of RCT studies (*n =* 6; 30%), whereas in single-component studies, the majority were quantitative non-randomized controlled trials or observational studies (*n =* 10; 42%).

There were no clear temporal trends in publishing dates, with 50% of the studies being published in the first half of our timeframe (2001–2008) and 50% published in the second half (2009–2016).

### Quality assessment of the studies (MMAT)

**Fig. 3:**
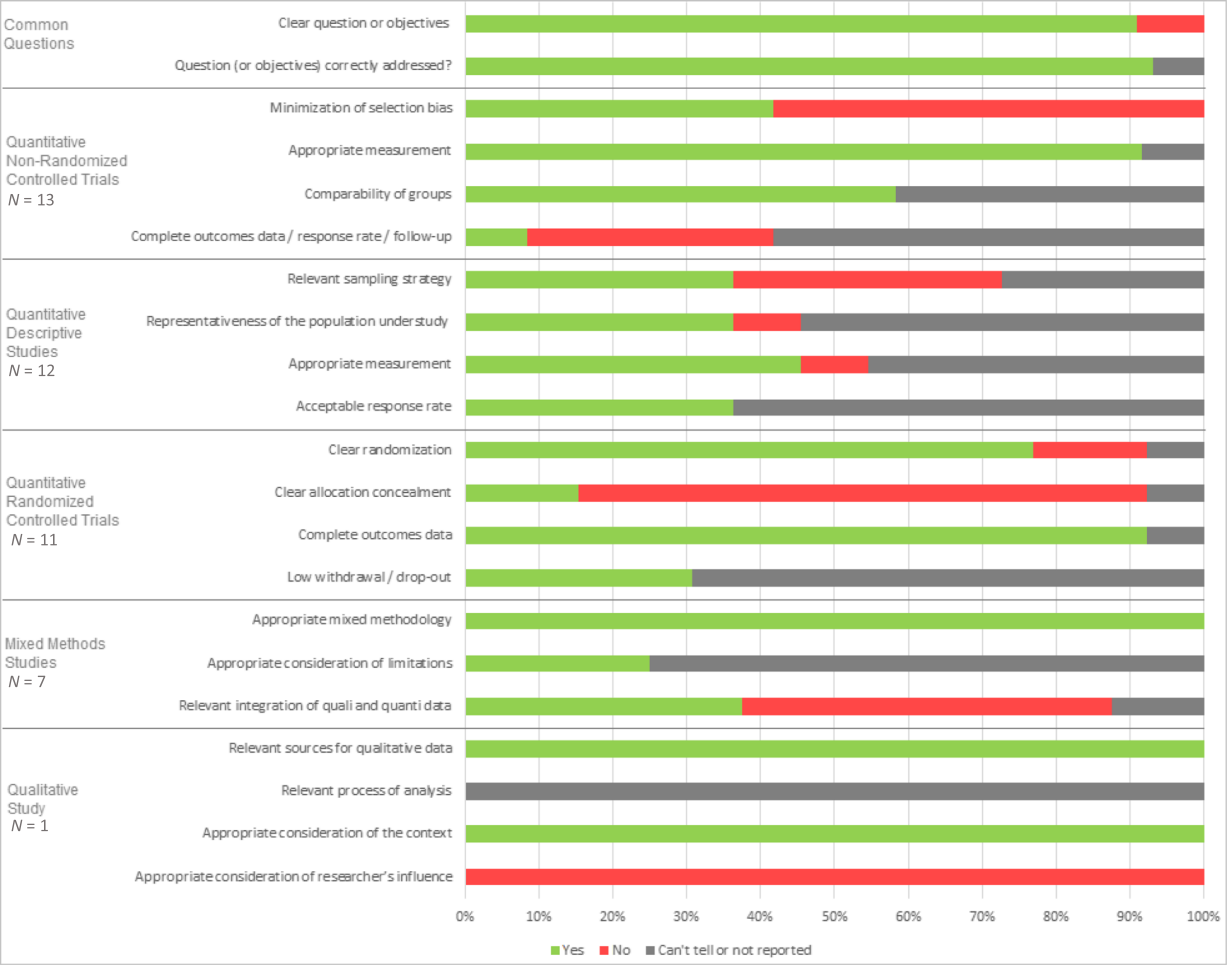
Quality assessment using the Mixed Method Appraisal Tool (MMAT). In green, percentage of studies answering “yes” to the question; in red, percentage of studies answering “no” to the question; in grey, percentage of studies answering “can’t tell” to the question

Overall, the included studies were assessed as having high risk for bias in the majority of the studies (Fig. 3). Four studies (9%) did not clearly state the study objectives and consequently, it was not possible to assess whether the objectives were correctly addressed [32, 41, 56, 62]. Four studies (9%) were rated as being of low risk for bias, with all indicators being positive (yes) [6, 30, 49, 51], while three (7%) were rated as very high risk for bias, with no positive indicators [56, 66, 67]. The remaining 36 (82%) studies were rated as moderate to high risk for bias, with at least one indicator positive [25–29, 31–48, 50, 52–55, 57–65]. When information was missing or lacked clarity in description, such as not reporting response rates or the presence of allocation concealment, the indicator was labelled ‘cannot be determined’.

### Description of the interventions

**Fig. 4:**
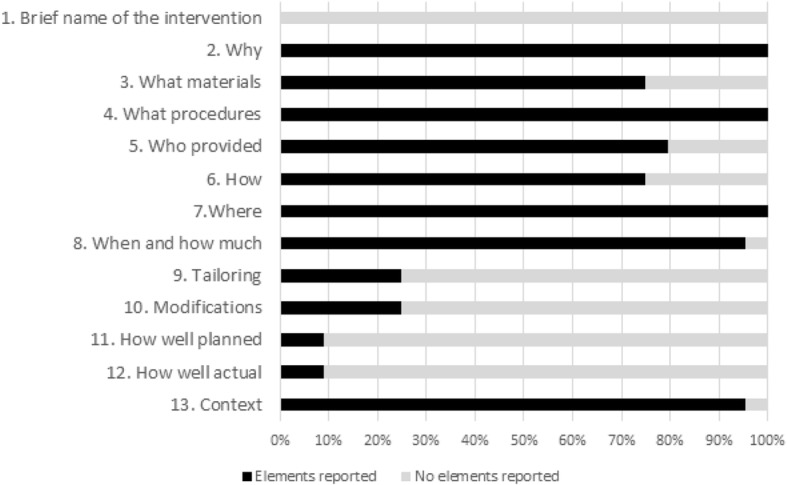
Description of interventions according to the TIDieR checklist. In black, percentage of studies reporting elements for each category; in gray, percentage of studies reporting no elements for each category

Figure 4 illustrates to what extent the interventions were described in each study, following the TIDieR checklist (see Additional file 3 for the complete extraction grid). Only a few elements were reported across all the studies: the reason for the intervention (‘why’), what the intervention was (‘what procedures’), the location (‘where’), the date and the frequency of the intervention (‘when and how much’), and some elements of context (e.g. geographic, climatic, previous outbreak events in the area). Other basic elements of the interventions were reported in 75% of the studies, such as: 1) exact materials used (‘what materials’), as in Winch et al., who provided an image of the poster they used during the intervention [34]; 2) description of the providers (‘who provided’), as in Healy et al., who clearly described the providers, AmeriCorps volunteers [62]; or 3) the mode of intervention delivery (e.g. person-to-person, group meetings) (‘how’), as in Vanlerberghe et al., who specified that: “During distribution, at least one person in every household received information on the use and maintenance of the insecticide treated materials through person-to-person communication” [50].

Eleven studies (25%) included information to explain potential customization or tailoring (or not) [6, 25, 29–31, 33, 43, 51, 53, 57], such as Andersson et al., who wrote that “each community chooses and implements its own mix of dengue prevention actions based on local vector reservoirs and community resources” [30]. Also, 11 authors (25%) provided information on modifications made due to external factors [6, 25, 36, 37, 42, 43, 46, 55, 57, 58, 65]. For example, Wai et al. described how a cyclone postponed all intervention activities, which occurred after a municipal campaign response including mass larviciding of water containers [58]. Lastly, regarding the process for evaluating the intervention, such as its fidelity (‘how well planned’ and ‘how well actual’ fidelity and adherence were assessed), four authors mentioned that the analysis was planned ahead of the intervention implementation [25, 30, 53, 58] and four authors provided information on fidelity [25, 53, 55, 57]. For example, Castro et al. explained that “in some intervention clusters, local actors introduced changes to the original design and, furthermore, the level of participation varied. This was documented in detail through process-oriented fidelity research that revealed important heterogeneity in the implementation” [53].

### Description of the process and transferability elements

Using the ASTAIRE checklist, we examined the availability of information for 23 elements related to the implementation process and transferability under four categories: population, environment, process, and elements needed for an intervention’s transfer (see Additional file 3, Fig. 5).

**Fig. 5:**
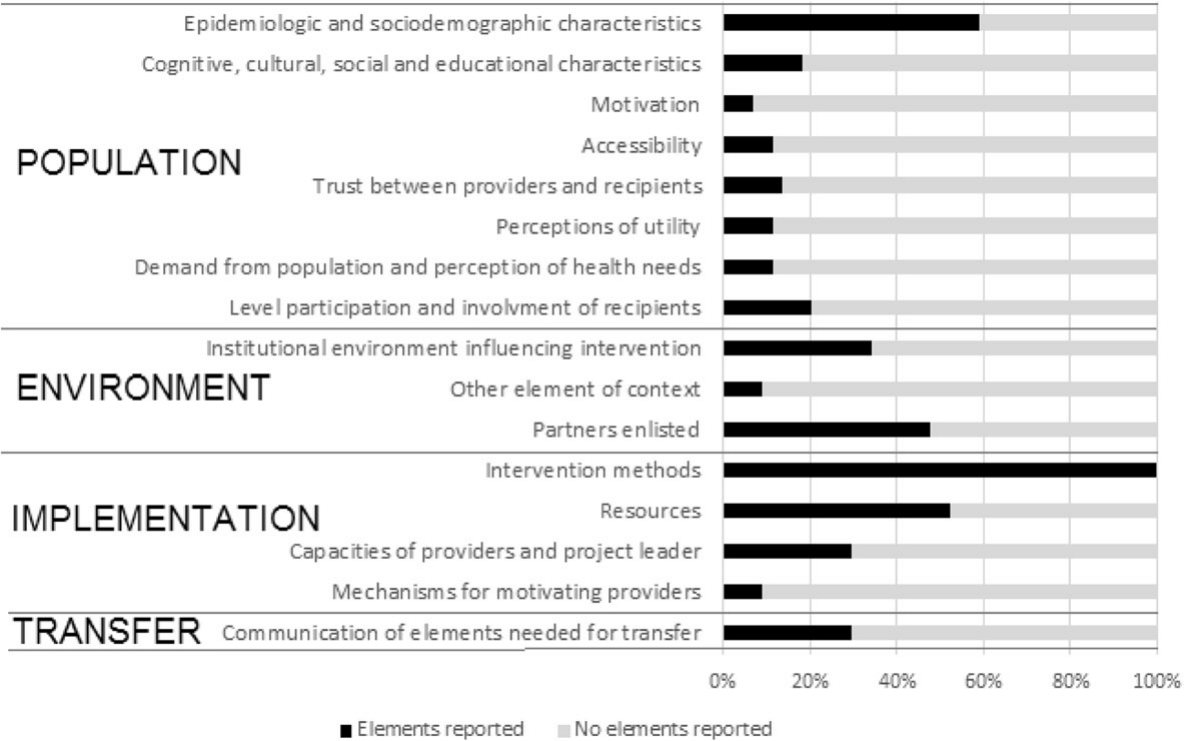
Contextual elements essential to intervention implementation and transferability according to the ASTAIRE checklist. In black, percentage of studies reporting elements for each category; in gray, percentage of studies reporting no elements for each category

Besides the intervention method, the elements most often reported were the epidemiological and sociodemographic characteristics of the population (60% of studies), the human and financial resources (50%), and partners enlisted (48%). Other aspects related to the implementation and transferability of these interventions were poorly described. For example, only five studies (11%) clearly provided information related to the perception of health needs within the community and how or whether these were taken into account [30, 31, 33, 35, 58]. Arunachalam et al. stated that there was a large demand for the water container covers from the community [51]. Fifteen studies (34%) noted that the institutional environment could influence the interventions [6, 25, 29, 31, 32, 34, 37, 45, 50, 51, 54–56, 58, 65]; for example, as Abeyewickreme et al. described: “a close collaboration was established between volunteers and local government authorities with a long-term view for the sustainability of activities when funding of the project ceases” [27].

Only two studies (5%) mentioned a theoretical framework to justify their approach [31]. Pengvanich provided a clear definition of the term ‘empowerment’ based on Wallerstein and Bernstein [68] as “a method […] where the group members are given necessary tools to identify the problem and its causes and are encouraged to find suitable solution by themselves” [31]. Subsequently, the author elaborated on the theory-driven design of the program, which was “specially designed based on the five-step learning process for empowerment (namely experiencing, identifying experience, analyzing, planning, and doing) which was created by Bishop [68, 69], and the participatory learning process which comprised experiential learning and group process” [31]. Sanchez et al. provided a conceptual model of their educational strategy to enhance community participation [33] and mentioned that the evaluation of the participation was based on the framework developed by Rifkin et al. [70].

### Types of interventions

Table 2 presents findings of all 24 single-component interventions, subdivided into five sections according to types of intervention activities: 1) chemical applications (*n =* 7; 29%), such as insecticide spraying or larvicide distribution in water tanks [25, 35, 41–44, 66]; 2) source reduction of breeding sites for mosquito populations via direct removal of stagnant water and/or through educational activities (*n =* 7; 29%) [26, 27, 36, 45, 61, 62, 67]; 3) traps (*n =* 4; 18%), which include mosquito and flea/rat traps [40, 46–48]; 4) nets (*n =* 3; 12%), which include bed nets, windows screens, and/or tanks covers [28, 49, 50]; and 5) biological agents (*n =* 3; 12%), such as fish that eat mosquito larvae or neem oil to repel sandflies [38, 63, 64].

**Table 2:** Main findings for all single-component interventions (*n =* 24)

Author Year	Country VBDs	Study design Sample size Duration	Components of intervention^a^	Effectiveness of intervention based on outcomes measures^b^	Challenges faced, lessons learned and/or recommendations
Chemical application (*n =* 7)					
Ocampo et al. (2014) [25]	Colombia Dengue	• Quanti non randomized • All 4800 street catch basins of the city (compared to a similar size city) • 18 months	• Productivity assessment of potential breeding sites indoor and outdoor (based on PPI) • Monthly application of pyriproxyfen in all street catch basins (designated as most productive breeding sites)	•↓ catch basins positivity for Aedes larvae after each monthly treatment (*P* < 0.001) • ↓ dengue incidence in Buga compared to Palmira (similar city) (rate ratio 0.19, 95% *CI*: 0.12–0.30, *P* < 0.0001) • No epidemic outbreaks occurred in Buga, while one occurred in Palmira	Involvement of field staff in designing and operationalizing entomological surveillance is important. Street catch basins are important breeding sites and their targeted control could help to decrease dengue transmission, especially in a context of hyper-endemicity such as Buga.
Ansari et al. (2001) [35]	India Malaria	• Quanti non randomized • 204 flats (= 992 people) in intervention and number of flats unknown (= 750 people) in control • 3 years	• Deltamethrin (insecticide) sprayed on both sides of cotton fabric curtains	• ↓ Indoor resting mosquitoes (87.9–93.7%, *P* < 0.05) and domestic pests (67.9–85.7%, *P* < 0.05) • ↓95.4% reduction in malaria cases per 1000 population	Insecticide-treated mosquito window and door curtains, along with legislative measures, may provide cost-effective (< USD 1) concurrent control of mosquitoes and other domestic pests.
Barrera et al. (2008) [41]	Puerto Rico Not VBD specific (*Ae. aegypti*)	• Quanti non-randomized • 1400 households in intervention area and 1500 households in control area (= 2900 households) • 3 months	• Containers likely to be aquatic habitats turned over and ones too large to be turned treated with 1 ppm methoprene • Spatial analysis using geographical information system to analyze the density of adult mosquitoes and detect hot-spots	• No differences in number of mosquitoes resting indoors • Spatial analysis revealed high-value clusters at 3 weeks (*Z* = 3.6, *P* < 0.01) and 5 weeks (*Z* = 7.55, *P* < 0.01) post-intervention • Septic tanks produced _~_ 18 000 *Ae. aegypti*/day, or 4.4 *Ae. aegypti* adults/person/day • Septic tanks contribute significantly to the maintenance of dengue virus endemicity	Control of *Ae. aegypti* in surface container is not sufficient to prevent dengue transmission. Visual detection of aquatic habitats of *Ae. aegypti* is not sufficient to assess adult mosquito productivity and thorough assessments of all public services can guide entomological surveys of *Ae. aegypti*.
Castro et al. (2007) [42]	Colombia Not VBD specific (*Ae. aegypti*)	• Quanti non- randomized • 200 households with permethrin and 126 for cypermethrin and unknown number of controls • 6 months	• Indoor ULV high-cis permethrin (Depe) (3 sprayings) and β-cypermethrin pyrethroids (1 spraying) in a smoke-generating formulation	• ↓ 82% mosquito density 24 h post 3rd spraying high-cis permethrin • ↓ 94% mosquito density 24 h post 1st spraying β-cypermethrin pyrethroids • No maintained reduction in mosquito density throughout 5 weeks	β-cypermethrin pyrethroids = potential alternative to organophosphate insecticides
Farajollahi et al. (2012) [43]	USA Not VBD specific (*Ae. aegypti*)	• Quanti non-randomized • 1250 parcels (homes + yards) in intervention area and 1064 parcels in control area (*n* = 2314 parcels) • 3 years	• Application of a novel adulticide DUET® between 1∶30 am–6∶30 am once or twice	• Single application at full label rate = ↓ 72.7 ± 5.4% mosquitoes recovered in traps. • Dual applications at mid-label rate spaced 1 or 2 days = ↓ 85.0 ± 5.4% mosquitoes trapped (more effective *P* = 0.003)	Night time ULV adulticiding is effective in reducing *Ae. albopictus* abundance at mid-label rate with dual application.
Perich et al. (2001) [44]	Honduras Not VBD specific (*Ae. Aegypti*)	• Quanti non-randomized • 6 houses in 5 city blocks (*n =* 30 households) • 4 months	• 4 treatment groups: ULV at front door, ULV in each room, thermal fog at front door or thermal fog in each room Chemical used?	• 24 h after: no mosquitoes collected in most houses of all groups • 4 weeks after: ↓ mosquitoes collected (*P* < 0.05) with both ULV spraying and thermal fog at the door • Starting 5th week: ↑ mosquitoes collected but still 2–4 times fewer than in control (differences with control during the 7 weeks post-intervention; *P* < 0.0001)	The use of ULV spray at front door is faster and more cost-effective since 1 spray operator could treat 75 houses in a day.
Cetin et al. (2006) [66]	Turkey Not VBD specific (*Culex pipiens*)	• Quanti descriptive • 7 septic tanks per group (*n =* 28 septic tanks) • 7 weeks	• Wet powder or granular formulation of diflubenzuron applied to groups of 4 septic tanks at different concentrations (0.01, 0.02, and 0.03 mg (AI)/liter)	• ↓ 100% adult *Cx. pipiens* in septic tanks with both formulations of diflubenzuron at 0.02 and 0.03 mg (AI/litre) (7, 14, 21 and 28 days post-treatment) • Water quality parameters influenced the field efficacy of larvicides	It is suggested that diflubenzuron may prove to be more useful in septic tanks for effective control of *Cx. pipiens* than currently used organophosphate larvicide.
Source reduction and/or educational campaigns (*n =* 3)					
Marquetti et al. (2006) [26]	Cuba Dengue	• Quanti descriptive • 156 households • 9 months	• All buildings inspected for water containers inside and outside of households: visual assessment of presence of organic matter and removal of infested water	• 5 troughs (0.71%) with an average of 6.4 pupae/trough. • 100% of positive troughs were dog troughs • Troughs were mainly placed at shadowed sites, in the backyard of houses, and had abundant organic matter	The population should be informed that drinking water should be replaced daily to decrease the number of breeding sites and at the same time improve the animals’ health.
Saurabh et al. (2014) [27]	India Dengue	• Descriptive • 351 participants • 2 weeks	• Individual health education (10 min) with pamphlet distribution giving information regarding source reduction (photographs and key messages)	• Practices regarding dengue prevention ↑ as compared to baseline measures: • ↑ 15.7% use of any methods for protection from mosquito bites during day time • ↑ 35% draining of unused tires • ↑ 26.7% draining of coolers	Individual health education was effective in improving the practice of source reduction in a community with poor knowledge of vector biting and breeding habits.
Alvarado et al. (2006) [36]	Colombia Malaria	• Mixed methods (descriptive) • 314 non-exposed and 347 exposed (= 661 women) • 3 months	• Community health workers trained 6 years ago to educate their community • Six interviewers visited households (semi-structured questionnaire) to do a post-intervention evaluation	• 14% in contact with intervention materials • ↑ attention to febrile episodes • ↑ use of bed nets (*OR*: 0.46; 95% *CI*: 0.23–0.93) • ↑ fumigation practice (*OR*: 0.38; 95% *CI*: 0.19–0.75) • ↓ Malaria cases (25% to 17%)	Accessibility and availability of material does not guarantee its use.
Tsuzuki et al. (2009) [45]	Viet Nam Not VBD specific (*Ae. aegypti*)	• Quanti descriptive • 26 premises in 6 areas (*n =* 122 premises) • 10 months	• All water storage containers indoors and outdoors inspected followed by installation of water supply system	• Drastic ↓ in the number of plastic buckets and water jars was reported between the first and second surveys • ↓ all entomological indexes (BI, CI, HI, PPI) in both intervention and control areas	Installation of a water supply system = ↓ number of water storage containers, such as plastic buckets and water jars. But remaining containers were still an important source of *Ae. aegypti* pupae.
Healy et al. (2014) [62]	USA Not VBD specific (*Ae. albopictus*)	• Quanti non-randomized • Around 800 households • 3 months	• Recruitment of volunteers from AmeriCorps and training (mosquito biology and role-playing techniques) • Community education included: [1] door-to-door active education (actively walking around the front and back yard with the resident, describing current and potential mosquito habitats + distributing educational material), [2] tire pick-up days, [3] trash can drilling days, and [4] media releases.	In both counties: • ↓ in unmanaged containers/home after intervention not sustained in following months but stayed under levels of control • spatial analysis: treatment sites = ↑ source reduction behavior vs continued ↑ in container habitats in control area • No clear effect on infestation levels	Recommended that an active education campaign could better promote source reduction behavior in the community to prevent peridomestic mosquitoes, rather than of passive education measure (distribution of pamphlets).
Bodner et al. (2016) [61]	USA Not VBD specific (*Culex pipiens*)	• Quanti RCT • 40 houses in 6 areas (*n =* 240 households) • 2 years	• Distribution of educational print materials included a calendar, a notepad, a flyer, and a magnet with pictorial and written mosquito education information	• ↓ concern for mosquito-borne illnesses • No effect on water-source and mosquito-infested containers	Print educational materials may have unintended negative effects on resident attitudes and household management of mosquito production.
Ensink et al. (2007) [67]	Pakistan Not VBD specific (*Ae. aegypti*)	• Quanti descriptive • 6 ponds in intervention and 6 ponds in control • 6 months	• Sanitization of waste ponds: reducing amount of floating matter + eliminating emergent vegetation + repairing cracks in the cement structure	•↓ 0% positive sample water for *Anopheles* and almost 0 for *Culex* in intervention ponds vs 19.2% and 34.0% respectively in control ponds	Recommended that vegetation management and maintenance of the concrete structures and waste inflow to waste stabilization ponds be improved in areas with a risk of mosquito-borne diseases.
Traps disposal (*n =* 4)					
Ratovonjato et al. (2003) [40]	Madagascar Plague	• Quanti RCT • 212 treated boxes in intervention area and 214 empty boxes in control area (=426 boxes) • 8 months	• Kartman boxes (wooden tunnel-like boxes having biscuits like baits containing a rodenticide and insecticide for fleas) placed	• ↑ Rats found dead: 968 in treated area vs 3 in control • Between J120–180: ↓ Prevalence of rats with fleas: 0% in treated area vs 60% in control area • Between J120–180: ↓ Cheopis index at 0 in treated area and 5.0 in control area	Kartman bait-boxes reached the rat borne and the vectors of plague found in urban area. This method can be used extensively both during epidemic and inter-epidemic contexts.
Barrera et al. (2014) [46]	Puerto Rico Not VBD specific (Ae Aegypti)	• Quanti non-randomized • 327 houses intervention and 241 houses control (*n =* 568 houses) • 1 year	• 3–4 CDC autocidal gravid ovitraps (AGO) per home (source reduction, larviciding, and oviciding before ovitraps installation)	• ↓ female *Ae. aegypti* (53–70%) • 3–4 AGO/home prevented outbreaks after rainy season	AGO traps were useful and inexpensive mosquito surveillance devices compatible with other control measures.
Nagpal et al. (2015) [47]	India Not VBD specific (*Ae. aegypti* and *albopictus*)	• Quanti RCT • Over 9000 houses in 3 districts • 3 phases over 6 years	• Meetings with communities; posters/pamphlets were distributed and then 2–4 ovitraps placed inside and outside of houses: half with attracticide C21, half without	• District 1: positive ovitraps with attracticide = 2.96% vs control = 1.41% (*P* < 0.05) • District 2: Positive ovitraps with attracticide = 1.25% vs control = 1.52% (*P* > 0.05) • District 3: Positive ovitraps with attracticide = 22.70% vs control = 21.38% (*P* < 0.05)	Chemical treatment using C21 attracticide has potential for surveillance and management of dengue and chikungunya mosquitoes. Recommendation for improvement of C21 delivery system, currently liquid formulation, but tablet formulation would be a better option.
Perich et al. (2003) [48]	Brazil Not VBD specific (*Ae. aegypti*)	• Quanti RCT • 30 houses intervention and 30 houses control in 2 cities (= 120 houses) • 3 months	• 5 lethal ovitraps (LOs) treated with deltamethrin outside and 5 LOs inside households (kitchen, bathroom, living room and each bedroom).	• CI ↓in both districts (4–5 vs 10–18) • HI ↓ in both districts (0.3–0.7 vs 8–10) • ↓ number of adult *Ae. aegypti* females indoors in one district (from 6.8 to 3.6/house) but not in the second (∼3/house)	LO is not designed to be sole dengue control vector; rather, meant to be integrated with other VBD control methods.
Distribution of nets (*n =* 3)					
Lenhart et al. (2008) [28]	Haiti Dengue	• Quanti C-RCT • 18 clusters in 2 areas of 387 and 630 houses (*n =* 1017 houses) • 1 year	• Distribution and installation of Olyset long-lasting insecticidal bednets to all houses (average 2/house)	• 1 month after: HI: − 6.7 (95% *CI*: − 10.6, − 2.7; *P* < 0.01); BI: − 8.4 (− 14.1, − 2.6; *P* < 0.01) and ↓ positive ovitraps (*P* < 0.01) • 5 and 12 months after: ↓ all indices compared to baseline control, including in control group • 12 months after: ↓ dengue serology from 33.7 to 18.5% (*P* < 0.01) • Acceptance and use: 96.5% houses still had ITNs and 52.2% participants slept underneath the day before	Insecticide-treated bednets had an immediate effect on dengue vector populations after their introduction, and over the next 5–12 months, the presence of ITNs may have continued to affect vector populations and dengue transmission.
Maciel-de-Freitas et al. (2011) [49]	Brazil Not VBD specific (*Ae. aegypti*)	• Quanti descriptive • Area of 867 households, each week 40 households sampled • 1 year	• 3 consecutive household pupal surveys in which all containers inspected for immature mosquitoes and classified according to type, with the most productive container types identified and covered using nylon net	• 1st survey: water tanks = most productive and thereafter covered. Rapid and intense ↓ in adult mosquito population density lasted only a few weeks • 2nd survey: ↓ infestation levels but dramatic ↑ in productivity in almost all container types. Metal drums were covered. Long-term ↓ in female mosquito density. • 3rd pupa • Survey: many fewer *Ae. aegypti* larvae and pupae collected but ↑ productivity in small containers	Large containers used by households for water storage were often key mosquito breeding containers where piped water distribution is irregular. Recommended that netting the most productive containers produces a drastic and long-term reduction in adult mosquito density without using chemical treatments.
Vanlerberghe et al. (2011) [50]	Venezuela Not VBD specific (*Ae. aegypti*)	• Quanti descriptive (within a C-RCT design) • 10 clusters: 5 urban (1742 houses) and 5 suburban (2359 houses) (*n =* 4101 houses) • 3 months	• ITMs distribution: curtains (up to a maximum of 5 curtains/house) and water jar covers + 1 person from each household received information about use and maintenance	• Months following the distribution: ↓ BI (IRR = 0.30, 95% *CI*: 0.22–0.49) and ↓ PI (IRR = 0.23, 95% *CI*: 0.14–0.37) • 18 months follow-up: ↑ gradually PI in suburban but not in urban clusters; BI remained consistently at 55% or more below pre-intervention levels in both settings	ITMs in housing can result in significant reductions in *Ae. aegypti* levels when dengue vector infestations are moderate, but the magnitude of the effect depends on the coverage attained, which itself can decline rapidly over time.
Biological agents (*n =* 3)					
Wagatsuma et al. (2009) [38]	Bangladesh Visceral leishmaniasis or kala azar	• Quanti RCT • 770 houses intervention and 780 control (*n =* 1550 houses) • 1 year	• Neem oil (insecticide) sprayed bi-weekly during summer and monthly during other months indoors of households	• No differences in sero-prevalence 1 year after (6.8% in intervention vs 4.8% in control) • No differences in sandflies population numbers • Number of engorged *P. argentipes* in control group was 7 times higher (*P* = 0.002)	Neem oil extract acts as a biopesticide and may be a good alternative for effective vector control of leishmaniasis. Other benefits include complex structure of azadirachtin, which makes it difficult for insects to develop resistance.
Tranchida et al. (2010) [63]	Argentina Not VBD specific (*Culex pipiens*)	• Quanti non-randomized • 3 groups of 4 drainage ditches (= 12 ditches) • 2 years	• Release of 1, 7, or 13 fish/m^2^ (predacious copepods) in drainage ditches	• ↓ 99% *Culex pipiens* larval stages within 15 weeks after introduction of the 13 fish/m^2^ and 22 weeks of 7 fish/m^2^	Predatory fishes are appropriate for long term control of *Culex pipiens* in human-made aquatic habitats but chemical, physical, and biological characteristics of the water are important determinants of the success of this method.
Fimia Duarte et al. (2009) [64]	Cuba Not specific	• Quanti non-randomized • 870 tanks intervention and 870 tanks control (*n =* 1740 tanks) • 1 year	• Larvivorous fish obtained from local rivers deposited in water tanks for non-human consumption (3 fish/container, 2 females and 1 male).	• 8 mosquito foci were prevented per 100 reservoirs treated with fish • Able to prevent 89% of breeding deposits.	Recommended to use local species of fish only.

^a^Abbreviations: *ULV* ultra low volume spraying, *ITNs* insecticide treated nets, *ITMs* insecticide treated material, *LLINs* long lasting insecticidal nets

^b^Abbreviations: *BI* Breteau index, *HI* house index, *CI* container index, *PPI* pupae/person index

Table 3 presents findings of all 20 multi-component interventions, divided into community-based approaches (i.e.*,* community mobilization) (*n =* 15; 75%) [29–34, 37, 51–58] or vertical approaches (*n =* 5; 25%) [6, 39, 59, 60, 65]. Vertical approaches refer to the more ‘traditional’ means by which most health programs and policies are delivered to populations; they do not involve the community in planning or designing the intervention [71]. In community-based approaches, on the other hand, community representatives and/or entire communities are involved in planning and/or designing the intervention [72].

**Table 3:** Main findings for all multi-component interventions (*n =* 20)

Author Year	Country VBDS	Study design Sample size Duration	Components of intervention^a^	Effectiveness of intervention based on outcomes measures^b^	Challenges faced, lessons learnt and/or recommendations
Community-based Approach (*n* = 15)					
Castro et al. (2009) [37]	Tanzania Malaria	•Quanti non randomized • 3 groups of 2 drains in 6 different area of around 300 houses each (*n* = around 1800 houses) •1 year	• Initial assessment of drains, • community sensitization and drain cleaning • evaluation.	•↓ malaria infection in intervention neighborhoods vs pre-cleaning period (*OR* = 0.12, 95% *CI*: 0.05–0.3, *P* < 0.001). •↑ risk of infection (*OR* = 1.7, 95% *CI*: 1.1–2.4, *P* < 0.001) in neighbourhoods under no intervention after cleaning •18 months after: only 1 drain still clean with continued maintenance efforts (other drain = lack of proper resources and local commitment limited success)	4 elements are needed for sustainable environmental management: i) political will and commitment, ii) community participation, iii) financial resources iv) and inter-sectoral collaboration.
Abeyewickreme et al. (2013) [29]	Sri Lanka Dengue	• Mixed methods (C-RCT) • 4 clusters of 200 households each in intervention and control (*n* = 1600 houses) •1 year	•Mobilization of the community and promotion of proper solid waste management at household level • Raising awareness on solid waste management among multi-stakeholders + improving garbage collection with the assistance of local government. •Awareness program and cleaning campaigns for school children in 8 schools. • Distribution of low-cost compost bins free of charge + 50% discount coupons given to buy additional bins • Qualitative analysis including: focus group discussions to plan and monitor interventions and to assess community mobilization; in-depth key informant interview to plan the intervention; stakeholder and gender analyses	•↑ regular of garbage collection by local authorities in and beyond intervention clusters • Women identified as key actors in the entire process of cleaning homesteads and solid waste management at household level. • On average, over 100 persons participated in each voluntary campaign to clean the outdoor environment (8 campaigns in total) • ↓ BI from 11.75 and 9.75 at baseline to 3.13 and 6.25 in the intervention and the control clusters respectively	The mobilization of the community was essential for the successful implementation and sustainability of the programmes. Coordination of local authorities and increased household responsibility is vital for effective and sustained dengue control.
Andersson et al. (2015) [30]	Nicaragua and Mexico Dengue	• Quanti C- RCT • 75 clusters in intervention (45 in Mexico and 30 in Nicaragua) and 75 control clusters (about 140 houses each) (*n* = around 19 000 houses) • 1 year	• All clusters = pesticide-free communities with ongoing standard vector control programs •3-steps mobilization program and each community chose and implemented individual prevention actions based on community resources: - permission asked from community leaders and engaged them in discussion of baseline evidence -Facilitators ran Intervention Design Groups to discuss results, costs, and specific prevention strategies -Volunteers received training as organizers and educators.	Relative risk reductions (95% *CI*) = Serology: 29.5% (3.8–55.3%) Dengue cases: 24.7% (1.8–51.2%) HI: 44.1% (13.6–74.7%) CI: 36.7% (24.5–44.8%) BI: 35.1% (16.7–55.5%) PI: 51.7% (36.2–76.1%)	Each site implementing the intervention in its own way has the advantage of local customization and strong community engagement. In contrast with current largely vertical programs distributing temephos or fumigating, dengue control should be rebuilt with fuller community engagement, collaboration with schools, and operational integration with local/municipal services
Pengvanich et al. (2011) [31]	Thailand Dengue	•Quanti descriptive •120 family leaders •8 weeks	• 2-day workshop for family leader about prevention and control of dengue and to provide a family leader’ activity manual. • Community leaders invited to discuss problems and obstacles involved in implementing dengue preventive programs and to suggest solutions. •Family leaders were asked to practice the knowledge gained and to record their behaviour in record form. • Weekly visit of volunteer for follow-up and collect data	• ↓ CI from 11.86 (SD: 10.93) to 0.24 (SD: 1.36) (*P* < 0.001) vs ↓ from 10.52 (SD: 10.31) to 6.81 (SD: 7.51) for control • ↓ HI from 62.31 (SD: 16.93) to 3.21 (SD: 2.46) (*P* < 0.001) vs ↓ from 60.63 (SD: 15.34) to 54.03 (SD: 12.51) for control	It was recommended that the family leaders when well trained are capable of carrying out the vector control protocol effectively.
Raju et al. (2003) [32]	Fiji Islands Dengue	•Quanti descriptive •100 premises per survey for a total of 9 surveys (*n* = 900 premises) • 9 months	•Radiobroadcasts and house visits for education of source reduction and awareness of dengue risks. • Community training for elimination of breeding sites. • communities encouraged to collect solid waste and trash in and around their houses and placed these along the roadside for collection and disposal	• Most abundant breeding sites = tyres and drums. Tins, flower vases, plant containers, shells and others were of secondary importance. • ↓ CI from 33 to 5% for rubber tyres and from 42 to 8% for drums. • ↓ BI (*Ae. aegy*pti) from 29 to 0. • ↓ BI (*Ae. albopictus*) from 44 to 4.	Dengue and vector control programmes must convince people to remove breeding habitats or, alternatively, to prevent *Ae. aegypti* from having access to water containers and other household items that are its potential breeding sites
Sanchez et al. (2008) [33]	Cuba Dengue	•Qualitative study •Involvement of 3 circumscription of around 1000 inhabitants each. No clear sample of interviews conducted •2 years	• Community working group (CWG) coordinate and implement actions to community level. • Learning group met quarterly to exchange and analyze experiences, and to develop proposals, reformulation of the work of the CWG. • Development of strategy of local communication and social mobilization: analysis of risk and local maps of transmission risks, development of targeted messages •Educational pamphlets and other educational material.	• ↓ 79% larval-pupal index • No cases of dengue detected • Qualitative analysis of satisfactory of participants and community leaders, empowerment of the communities highly appreciated	Educational activities are effective to promote behavior change and raise awareness.
Winch et al. (2002) [34]	Puerto Rico Dengue	• Mixed methods (quanti non randomized) • No clear sample size • No clear duration	•Four community-based programs: -Head Start federal program for low income preschool children (classroom and community activities) -Public school program with preventive in the regular fourth grade social science curriculum -Posters and televised public service announcements -Children’s Museum exhibit on *Ae. Aegypti* • Semi-structured interviews and focus groups with parents, teachers, and officials + surveys on knowledge and exposure administered in the classroom to children and at home to the parents	• ↑ overall dengue-related knowledge associated to exposure of children • ↑ tires protected from mosquitoes • ↑ parent-child communication about dengue • ↑ use of aerosol insecticides with the parent’s exposure to Posters. • Overall, programs ↑ awareness and some behavior change • no differences on larval infestation • no differences with numbers of disposable containers	The results suggest that schoolchildren do communicate with their parents about dengue prevention, and that school programs can increase parental involvement in dengue control, but more specific messages about the behaviors to be performed need to be directed directly at parents
Arunachalam et al. (2012) [51]	India Not VBD specific (*Ae. Aegypti*)	•Mixed methods (C-RCT) •10 clusters of 100 houses each in intervention (*n* = 4639 inhabitants) and control (*n* = 4439 inhabitants) •10 months	• 17 meetings with multiple stakeholders to discuss vector control activities + mobilization of women’s self-help groups for clean-up campaigns. •Involvement of community to distribute water container covers and culturally and linguistically relevant health education materials. •Mobilization of school children regarding dengue prevention and environmental sanitation. •Fostering of waste disposal and recycling measures to eliminate small discarded containers.	• ↓ PI to 0.004 from 1.075 (*P* = 0.020). • ↓ HI to 4.2% from 19.6%; • ↓ CI to 1.05% from 8.91%. • ↓ BI to 4.3 from 30.8 • Knowledge of respondents about vector and disease transmission was enhanced significantly (*P* < 0.05).	A community-based approach and alliance with multiple stakeholders led to a substantial reduction in dengue vector density. The most prominent benefit was the satisfaction created by ‘working together’, expressed during the in-depth interviews.
Caprara et al. (2015) [52]	Brazil Not VBD specific (*Ae. Aegypti*)	•Mixed methods (C-RCT) •10 clusters of 100 houses in each intervention and control groups (*n* = 1000 houses) •5 months	•Community workshops + community involvement in clean-up campaigns •Covering elevated containers and in-house rubbish disposal without larviciding + source reduction of productive container. •Mobilizing schoolchildren and senior inhabitants •Distributing health education materials •Establishing partnerships with inter-sectorial groups •Requested the regional secretariat for a truck for waste collection	•↓ vector population via 100% elimination of most productive container types in all visited houses •↑ knowledge of dengue and willingness to participate in preventive actions. • Social participation was heterogeneous and shaped by historical and actual community dynamics	The results showed the effectiveness of the intervention package in comparison with the routine control programme. It is recommended that such a participatory eco-health approach offers a promising alternative to routine vector control measures such as larvicide treatment or space spraying without any social participation.
Castro et al. (2012) [53]	Cuba Not VBD specific (*Ae. Aegypti*)	• Quanti RCT • 16 clusters intervention and 16 controls of 390 houses each (*n* = 780 houses) • 2 years	• Creation of Management Group with researchers and community leaders; • Modification of routine entomological surveillance to optimise the relationship between the vector control programme and the communities • Community Working Groups (CWG) created for capacity building rooted in popular education theories; • CWG initiated the community work for dengue vector control organised as a cycle of sequential phases developed at circumscription level	• ↑ 36.2% adequate *Ae. aegypti* control practices at household level (vs no change for control; *OR*: 3.23) • ↑ 52.8% knowledge of breeding sites (vs 27.5% for control; *OR*: 1.50) • ↑ 316% risk perception of contracting dengue fever (vs 211% for control; *OR*: 1.63) • BI ↓ 53% (95% *CI*: 22–92%)	The empowerment strategy increased community participation and effectiveness of intervention than only routine *Ae. aegypti* control.
Pai et al. (2006) [54]	Taiwan Not VBD specific (*Ae. Aegypti*)	•Quanti descriptive •90 households, 190 respondents •5 months	• Evaluation post-intervention of a short-term community-based cleanliness campaign (no details) • Ovitraps placed indoors and outdoors for 5 days + knowledge and behaviour survey	• Ovitrap index ↓ from 66.7 to 39.3% 3 months after the campaign (*P* < 0.05). • Significant improvement in behaviour of source reduction after campaign (*P* < 0.05).	The study recommended that a short-term community-based cleanliness campaign is an effective alternative to rapidly reduce the sources of dengue vector at the onset of a new epidemic.
Qunitero et al. (2015) [55]	Colombia Not VBD specific (*Ae. Aegypti*)	• Quanti C-RCT • 10 clusters of around 100 houses each in intervention and control (*n* = 1825 households included) •6 months	• 3 LLINs for windows and 1 LLINs for door distributed/households (1st phase) • Mean 1.2 lid distributed to cover water containers (2nd phase) • Household heads interviewed by field staff + focal group discussions with local people organised to assess coverage, use and satisfaction	• LLINs alone: ↓ BI from 14 to 6 vs 8 to 5 for control (*P* = 0) • + containers lids: ↓ PI 71% vs 25% for control (*P* = 0.01). • Mainly, participants were impressed by the number and diversity of dead insects below curtains and they would recommend the interventions to others	The results indicate that the intervention package can reduce dengue vector density. Successful and adequate use of the intervention packages should be enhanced through appropriate social mobilisation to achieve long-lasting behavioural change.
Toaliu et al. (2004) [56]	Vanuatu island Not VBD specific (*Ae. Aegypti*)	•Quanti descriptive •1500 inhabitants • 5 year	•Identification of partners for assisting in advocacy and knowledge dissemination • Formation of a community committee including chief, youth and women representatives • Series of meetings and workshop for the community on dengue fever, its prevention and control. • Play performed during workshop and in local school involving the audience walking around to identify and destroy breeding habitats, either by complete removal or by application of temephos + distribution of leaflets with key messages from the play • Same theatre company developed TV/radio spots • Development of videos and school handbook on key messages of dengue prevention • Mobilization for waste removal (garbage bags; old car tyres management; lories for waste collection).	• ↓ epidemic outbreaks after intervention and only around 100 cases of dengue were recorded with no mortality	Vertical programmes run by the health sector without community participation will struggle to be successful. Instead, programmes that develop horizontal partnerships, with community committees, will encourage community action and lead to more successful and sustainable outcomes.
Vanlerberghe et al. (2009) [57]	Cuba Not VBD specific (*Ae. Aegypti*)	•Mixed methods (C-RCT) •16 clusters of 2000 inhabitants each in intervention and control (*n* = 6400 inhabitants) • 1 year	• Routine vector control programmes (entomological surveillance, source reduction, selective adulticiding, and health education) implemented in all clusters •Discussion with relevant local stakeholders and formation of local steering committee • Creation of formal task forces (community working groups) at grassroots level to secure community involvement in environmental management •Each community working group carried out a situation assessment with the community, identified local needs and priorities for environmental and dengue control, and elaborated action plans •Action plans varied between circumscriptions including activities such as [1] locally designed social communication intending to mobilise the population and change behaviour; [2] negotiations with community and governmental inter-sectoral groups to eliminate environmental risks outside households; [3] surveillance of environmental risks with locally produced and periodically updated maps; [4] visits by teams of community members to houses with repeated Aedes infestation.	↓ Entomological indices in intervention vs control clusters: • HI: 0.49 (0.27 to 0.88) • PI: 0.27 (0.09 to 0.76). • Overall community involvement assessed as “fair” (average overall score 3.34) compared with almost non-existent before intervention	• A community based environmental management strategy embedded in routine control program is effective. • Involving the community takes time and is not a spontaneous activity. A suitable formal organisation must be identified or set up to guide the community involvement strategy and members of these organisations need training
Wai et al. (2012) [58]	Myanmar Not VBD specific (*Ae. Aegypti*)	•Mixed method (C-RCT) •13 clusters of 100 households each in intervention and control (*n* = 2600 houses) •9 months	• Eco-friendly multi-stakeholder partner groups (EFG) (led by ward authorities + midwives, members of Maternal and Child Welfare Association, trusted persons and school teachers) organize/ mobilize householders to accept interventions •10 ward-based volunteers selected by EFG to participate actively in controlling dengue vectors and visiting houses •Vector control tools applied according to the type of container and peoples’ preferences: chemical (insecticide), mechanical (cover nets), biological (dragon fly nymphs) + waste-collection bags for removing discarded small containers. •intensive awareness-raising campaign for the local communities through group discussions • Focus group discussions to underscore satisfaction and opinions + formal household survey for the acceptance of intervention tools	• Combined measures most frequently favoured (44.8%), then chemical (34.2%) and mechanical measures (16.5%). • ↑ people’s awareness of appropriate vector control options for specific containers as well as positive attitudes towards joint actions • ↑ householders’ responsibility in managing vector breeding sites • Massive larviciding programme in response to cyclone struck happened during the study and the intervention programme was as good in ↓ vector densities as the massive larviciding in the routine service areas: ↓ PPI from 0.34 to 0.23 (32% reduction) in intervention clusters and from 0.33 to 0.15 (54.5% reduction) in routine service clusters.	In terms of sustainability and empowerment of communities and other stakeholders, the partnership approach with targeted containers interventions was found to be superior to the vertical approach. The efficacy of the intervention was equivalent to the massive vertical larviciding programme in the aftermath of cyclone
Vertical Approach (*n* = 5)					
Moosa-Kazemi et al. (2007) [39]	Iran Anthropo-notic cutaneous leismaniasis (ACL)	• Quanti RCT • 3 groups of 160 houses (*n* = 380 houses) • One year	• Distribution of bed nets and curtains impregnated or not with deltamelthrin SC 5% • Health education messages were relayed to encourage use of bed nets and curtains, to explain the role of sand flies in transmission of ACL during the distribution via face-to-face and focus groups. • Schools visited, and teachers urged to educate on the importance to protect themselves from sandfly bites	• ↓ ACL rates in impregnated area (*P* = 0.02) vs non-impregnated and control areas • Use of nets and knowledge over 80%	Personal protection is an effective and sustainable means of preventing and controlling ACL and can reduce dependence on insecticides
Gurtler et al. (2009) [6]	Argentina Dengue	• Descriptive • City wide (168 603 houses visited; 120 000 surveyed; 37 000 treated) • 5 years (14 cycles of 4 months)	• Pre-interventio*n =* media campaign to inform the community • House visit = treatment of water containers with temephos + manually source reduction (removal of containers) + householders encouraged to maintain appropriate containers management • Inspection and larvicide efforts in cemeteries and used tire lots	• ↓ Dengue cases (by DEN-1): 10.4 per 10 000 in 2000 to 0 per 10 000 in 2006 • ↑ Dengue cases (by DEN-3): 4.5 per 10 000 in 2007 • ↓ BI and HI in nearly all focal cycles compared to pre-intervention but failed to keep them below the desired target levels	(i) achievement of sustained community acceptance; (ii) most likely averted new dengue outbreaks between 2003 and 2006, and (iii) limited to alarge extent the 2007 outbreak of DEN-3 in an immunologically naive population.
Abramides et al. (2011) [65]	Spain Not VBD Specific (*Ae. Albopictus*)	• Quanti non randomized • 6 areas of 100 to 470 houses each (*n* = 2104 houses visited phase 1 and *n* = 1000 houses visited phase 2) • 2 years	• House visits to reduce container habitats and education about VBDs + larvicide treatment for containers that could not be emptied; • Larvicide treatments in scuppers, water tanks and street drains containing stagnant water; • Sanitization of municipal sites and wooded terrains, with removal of uncontrolled rubbish dumps • Insecticide sprayed monthly on vegetation in public gardens	↓ mosquito eggs (*P* < 0.05)	Combination of the 4 strategies was effective in reducing the number of eggs and a high level of public cooperation was obtained. Door-to-door communication programme can have a long-term effect on the behaviour of the population.
Che-Mendoza et al. (2014) [59]	Mexico Not VBD Specific (*Ae. Aegypti*)	• Quanti C-RCT • 10 clusters of 100 households each in intervention and control (*n* = 2000 houses) • 2 years	• Installation of Duranet LLINs treated with 0.55% alpha-cypermethrin • Person-to-person information on use and maintenance of LLINs • Water tanks and drums/barrels treated with larvicide Natular DT.	• Only LLINs: ↓ infestation with *Ae. Aegypti* at 5 and 12 months after • LLINs + water tanks treated: ↓ infestation with *Ae. Aegypti* at 18 and 24 months after	Combination LLIS fitted to external windows and doors and targeted treatment of the most productive Ae. aegypti breeding sites can impact significantly on dengue vector for up to 24 months
Espinoza-Gómez et al. (2002) [60]	Mexico Not VBD specific (*Ae. Aegypti*)	• Quanti RCT • 4 groups of 45 to 49 households each (*n* = 187 houses) • 6 months	• 1 group = Educational campaig*n =* 3 visits per house (principally to housewives) + group meetings + video + small gift (sweets, stickers and calendars about dengue) • 1 group = Malathion ULV spraying + routine vector control operations (water containers treated) • 1 group with both and 1 control	• Overall CI ↓: 0.97 to 0.77 • More ↓ for educational group (F = 8.4, *P* < 0.005) than malathion spraying group (F = 0.38, *P* > 0.5) • Combination = discrete negative interaction (F = 6.52, *P* < 0.05) • No differences in Knowledge And Practice indicator (F = 1.14, *P* > 0.1)	Inter-sectorial integration with the community for an educational campaign is an effective measure and use of chemicals (ULV) should be reserved for epidemical outbreaks.

^a^Abbreviations: *ULV* Ultra Low Volume spraying, *ITNs* Insecticide Treated Nets, *ITMs* Insecticide Treated Material, *LLINs* Long Lasting Insecticidal Nets;

^b^Abbreviations: *BI* Breteau Index, *HI* House Index, *CI* Container Index, *PI* Pupae / Person Index;

### Evaluation of intervention effectiveness

Regardless of the type of intervention, 42 studies (95%) used at least one of the following entomological indices to assess intervention effectiveness: container index (CI: percentage of water-holding containers infested with larvae or pupae) in 24 studies (55%) [25, 26, 28–32, 34, 37, 41, 45, 47–49, 51, 52, 55, 58–61, 63–65, 67]; Breteau index (BI: number of positive containers per 100 houses inspected) in 15 studies (34%) [6, 28–30, 32, 34, 42, 50–53, 55, 57–59]; house index (HI: percentage of houses infested with larvae and/or pupae) in 13 studies (30%) [6, 28–31, 33, 35, 48, 51, 52, 57–59]; pupae per person index (PPI: number of pupae per number of inhabitants) in nine studies (20%) [29, 30, 49–52, 55, 58, 59]; traps positivity (percentage of traps found positive) in seven studies (16%) [28, 38–40, 43, 46, 54]; indoor resting adult mosquitoes (based on manual collection with vacuum) in six studies (14%) [41–44, 48, 66].

Nearly half of the studies (*n =* 21; 48%) included at least one of the following population-based indicators: assessment of concern or perception changes (*n =* 8; 18%) [30, 33, 37, 52, 53, 55, 58, 61]; willingness or actual participation or degree of involvement in the intervention (*n =* 7; 16%) [29–31, 51–53, 57], as well as use of the tools provided (e.g. nets, educational activities) (*n =* 6; 14%) [28, 36, 37, 39, 50, 55]; changes in behaviours, such as self-reported or objectively measured source reduction or healthcare seeking during febrile episode (*n =* 7; 16%) [27, 34, 36, 53, 54, 60, 62]; knowledge and misinformation assessment (*n =* 7; 16%) [27, 34, 39, 51, 53, 54, 60]; and acceptability elements (*n =* 4; 9%) [33, 35, 52, 58].

Only 25% (*n =* 11) of the studies that used epidemiological data collected primarily serological data or data from local surveillance systems to assess the effects of the interventions on specific VBDs. It was not always clearly stated whether the cases were clinical or laboratory confirmed cases.

Half of the studies (*n =* 22, 50%) [26, 27, 32, 38, 40–49, 56, 59, 62–67] used indicators only from one of the above categories (entomological, population-based, or epidemiological), while the other half (*n =* 22, 50%) used indicators from more than one category [6, 25, 28–31, 33–37, 39, 50–55, 57, 58, 60, 61]. The majority of the single-component intervention studies (*n =* 18, 75%) used indicators from only one category [26, 27, 38, 40–49, 62–64, 66, 67], while 16 (80%) of the multi-component intervention studies used indicators from multiple categories [6, 29–31, 33, 34, 37, 39, 51–55, 57, 58, 60].

Only eight studies provided information regarding the costs of the interventions [37, 39, 46, 50–52, 55, 67], and no study included a complete economic evaluation, such as a cost–benefit evaluation. Mostly, authors included minimal information, such as Arunachalam et al., who wrote: “Netted frames of three sizes (small, medium, and large) were made locally by sub-contractors and the cost was USD 8 per cover” [51], or Caprara et al., who included human resources costs in the intervention cost estimate: “the total costs of the intervention was USD 18.89 per house” [52].

Almost all the studies (95%) reported at least one positive indicator of intervention effectiveness. The only two studies reporting null or negative results were single-component interventions. Barrera et al. [41], in Puerto Rico, reported no effect on the density of adult mosquitoes resting indoors with the intervention consisting of source reduction of breeding sites and application of larvicide. The authors later showed that septic tanks (not targeted by the original intervention), significantly contributed to the maintenance of dengue virus endemicity in the region, with an estimated productivity of 4.4 *Aedes aegypti* adults/person/day (based on three persons per household). Bodner et al. (2016) observed a negative effect of their educational intervention, characterized by a decrease in concern for VBDs with no change in mosquito infestation rates or breeding site rates post-intervention. The intervention occurred in the US and consisted of distributing educational print materials including a calendar, notepad, flyer, and magnet, all with pictorial and written mosquito educational information. The authors suggested this print-focused educational campaign was insufficient to reliably motivate resident-based mosquito habitat reduction and may even have had the unintended opposite effect, making residents less concerned. The lack of active community involvement in the campaign and the authors’ inability to evaluate whether recipients had actually read the materials were possible explanations for these unexpected results [61].

### Challenges faced, lessons learned, and recommendations

Papers describing multi-components interventions were more to include descriptions of the challenges the research team encountered, in contrast to the single component intervention studies, which is likely due to their complex designs. For Gürtler et al., despite positive indicators of effectiveness, the intervention failed to maintain larval indices below targeted levels, for which they suggested seven possible reasons: 1) incomplete surveillance coverage; 2) limited residual efficacy of temephos; 3) permanent sites for mosquito breeding due to lack of change in the management of large containers for permanent water storage; 4) very favourable climatic conditions for *Ae. aegypti*; 5) limited source reduction efforts; 6) lack of regular perifocal residual spraying with insecticides; and 7) lack of adequate, sustained community participation beyond mere acceptance of regular control measures, for which there were high levels [6]. These described challenges were not isolated to Gürtler et al. One of the most commonly cited difficulties involved the sustainability of the interventions without the support and resources of the research teams [29]. This is difficult, as explained by Quintero et al., that despite initial success, intervention benefits can be forgotten and use of tools (e.g. nets) are discontinued [55]. There is an important need for continual encouragement and monitoring of the implemented programs [73]. Sustainability is also jeopardized by the need for significant investments of both human and financial resources for successful interventions, particularly for community-based interventions. This type of intervention requires increased time and resources compared to conventional institution-based interventions due to the longer socialization and negotiation processes needed to implement the intervention, to achieve social participation, and to respond to community expectations [55].

It cannot be expected that community participation in vector control interventions is simple. As discussed by Caprara et al., the social participation of subjects and groups is often heterogeneous and shaped by historical and present-day community dynamics [52]. For example, in their intervention in Brazil, “social participation was fragile in locations with nonexistent community organizations or in neighbourhoods with either a history of violence or very well off and privileged groups” [52]. Intervention protocols that engage leadership and community members in discussing evidence and defining local strategies are a promising starting point for a wide range of settings to ensure community participation in vector control activities [30]. Sites that implement interventions with their own approach have the advantage of local customization and strong community engagement, as demonstrated in the Camino Verde intervention in Nicaragua and Mexico [30].

In addition to community participation, achieving and maintaining field staff motivation is a major challenge in vector control activities. As found by Ocampo et al. [23], resistance during intervention implementation can arise among field staff: “Although we found that the field technicians initially objected to counting pupae, once they were aware of the low productivity of mosquitoes in houses, they began to understand the importance of obtaining these data.” Thus, a lesson learned from this intervention in Colombia was “the importance of engaging field staff in designing and operationalizing entomological surveillance. At first, counting pupae and increasing the number of houses to be sampled was strongly opposed by technicians. During the training activities, an agreement was achieved to classify visually the number of pupae but other methods with a perceived lower workload could be developed.” The coordination of local authorities, along with increased household responsibility for targeted vector interventions, is vital for effective and sustained dengue control, according to Abeyewickreme et al. [29]. Both Caprara et al. and Andersson et al. recommended expanding the coordination beyond local authorities, to include other sectors for sustainability. These sectors include education, local/municipal services such as water supply, garbage disposal, sanitation and street cleaning, culture, tourism, transport, construction, and public safety [30, 52].

A unique challenge was reported by the only study with negative results. Those authors concluded that print education materials may have had unintended negative effects on residents’ attitudes and household management of mosquito production, which resulted in no behavioural changes and decreased concern around VBDs [61]. This was one of the few reviewed studies conducted in a high-income country (US), where population characteristics would be very different from those in the other studies; however, they were not detailed in this article. Anecdotally, the authors reported that when some of the residents understood that the most important mosquito-borne threat in the region under study (Washington, DC, and Maryland) was West Nile virus, they appeared less concerned about mosquito vectors, relative to other diseases with more negative media attention and greater public health impacts, such as HIV or Ebola [61]. Moreover, Alvarado et al. (2006), in a post-intervention evaluation of population education, noted that accessibility and availability of material does not guarantee its use [36]; this might be one explanation for the negative results obtained by Bodner et al. [61], who were unable to evaluate whether people had actually read the educational materials provided.

## Discussion

This review emphasizes the need for higher quality research and improved reporting of interventions for VBDs. The trend towards more multi-component and community-based interventions is promising for enhanced effectiveness and sustainability of vector control strategies, although such interventions present important challenges that need to be considered from the outset.

Overall, the included studies were judged to be of high risk for bias, with the limited information provided. Context is a key as it is essential for understanding the elements needed to ensure successful interventions and to interpret the failures of previous interventions, which should be considered by researchers and implementers [74]. In vector control strategies, communal water supply or garbage collection services are significant determinants that require consideration in the intervention and evaluation. Using checklists such as TIDieR and ASTAIRE would be valuable to guide authors towards thorough and standard reporting of interventions. Given the large heterogeneity of interventions, study designs, contexts, and indicators, it was not possible to pool the findings for an average measure of intervention effectiveness in the framework of a scoping review.

Most of the studies measured their success using entomological indicators with only 25% using human morbidity indicators, despite the uncertain relationship between entomological indicators and the relative human morbidity indicators [75]. Reduction of vector populations is essential, but even significant reductions do not prevent epidemics or endemicity [76]. Thus, epidemiological assessment is essential to objectively evaluate an intervention’s effectiveness in reducing disease burden. Community-based interventions often provide more complex evaluations based on a greater diversity of indicators, including acceptability, use of tools, behaviour changes, and/or knowledge improvement. As vector control interventions are implemented in complex contexts, their evaluation strategies should capture all the components needed to objectively evaluate real world interventions [77].

The sustainability of vector control interventions is a key factor when attempting to scale-up research projects towards large-scale programs or policies [78]. Intervention sustainability was a critical challenge highlighted in several of the reviewed publications. The limited durations of follow-up and the lack of rich, qualitative data made it very difficult to evaluate intervention longevity or to understand not only the social and cultural determinants of interventions, but also their implementation processes, adaptability, and customization [79]. Thus, more implementation research is needed in VBD, including qualitative research methods with longer follow-ups to collect information on processes and sustainability.

The multi-component community-based interventions included in this review showed promising results, with interventions producing larger effects than standard vertical vector control programs in terms of both reductions of mosquito populations and increased sustainability [29, 30, 51–53, 57, 58]. However, involving the community requires time and resources [57]. Suitable organizations must be identified or created to guide the community involvement strategy and members of these organizations need training and support. The use of intervention packages should be enhanced through appropriate social mobilization to achieve long-lasting behavioural change [55] and with the active involvement of health promotion experts to inform to how change behavior.

Theoretical frameworks are essential when designing and implementing health education programs, given the need to understand psychosocial factors underlying individual and community-level decisions and behaviours [80]. Community-based programs show potential; however, the link between program outputs and vector presence is complex and generally unclear to which elements or specific actions an effect should be attributed [53]. Despite this, making community-based programs more flexible and adaptable is important for the future success of vector control strategies [78]. It is vital in the planning stages to identify the appropriate blend of core strategy components required to maintain effectiveness and the components that can be adapted and tailored to local conditions.

The interventions were largely focused on solutions to minimize vector breeding sites with only three studies focused on sanitation interventions: domestic septic tanks (Turkey) [66], water supply systems installation (Viet-Nam) [45], and sanitization of waste stabilization ponds (Pakistan) [67]. It is imperative to understand how improved sanitation infrastructure, including a stable potable water supply, results in reductions in vector breeding habitat and human morbidity. Moreover, including sanitation as an intervention would lead to more integrated disease management, as other pathogens (e.g. bacteria, parasites, viruses) and vector populations would be reduced.

Importantly, only a few non-mosquito vector studies were included (sandflies and fleas) with dengue predominantly represented, revealing substantial gaps in VBD research in urban contexts. Another significant research gap is the under-representation of Africa as only two African-based studies were included: Madagascar [40] and Tanzania [37]. West Africa, where countries are among the poorest and health problems are major [81], is completely absent in this review. Historically, VBD research in Africa has been dominated by malaria, which is considered a rural disease, and the significant malaria burden in Africa has often eclipsed other febrile illnesses [82, 83].

### Limitations of the study

Despite our best efforts, we were unable to retrieve the full texts of 14 potentially eligible articles (based on title screening). We may have also missed relevant publications in languages not included in our review. In addition, the fact that our inclusion criteria focused exclusively on urban areas may explain the smaller number of studies included in our review from Asia and Africa compared to the Americas. It should be noted that the most urbanized regions are in North America (82% living in urban areas in 2014), Latin America and the Caribbean (80%), and Europe (73%). In contrast, Africa and Asia remain mostly rural, with 40% and 48% of their respective populations living in urban areas [10]. The present review is also limited to published material and publication bias may influence some results presented in this review.

### Implications for future research

Several knowledge gaps were identified in this review that need to be addressed in future research. Broad community participation and social mobilization are central to the success of complex health interventions and community-based interventions are promising and should be encouraged [84].

However, the complexity of community-based interventions and questions of sustainability of community participation requires a comprehensive evaluation strategy with both quantitative and qualitative data collection. Given that few studies in our review addressed the long-term sustainability of community-based interventions, several questions remain and more research is needed [85, 86]. Increased eco-health research is needed to understand ecologically sustainable measures, such as non-impregnated nets to cover water containers and non-toxic alternatives [87], given the increased concerns of insecticide resistance [7] and human health consequences from acute and chronic exposure to chemical agents [88–91].

There is a critical need for researchers to report the methodology and the context of interventions clearly and completely to enhance the comparability of studies and the transferability of effective interventions to other locations and contexts. Checklists such as TIDieR [23] and ASTAIRE [24] are valuable standardized tools whose use should be encouraged and could conceivably become a publication requirement. Finally, there was minimal mention of theoretical frameworks being used for interventions, nor of the associated tools used, such as educational materials or workshops. Both researchers and stakeholders would benefit from theory-based approaches (and evaluation) to VBD interventions, which would be helpful in identifying successful and unsuccessful elements of an intervention [92].

### Implications for public health policy and/or practice

Several studies concluded that single-component interventions, such as use of insecticides, must be considered as one of the available measures for VBDs prevention, but not the only one [49]. Multiple-component community-based interventions, such as environmental management, education, and social mobilization, are promising in their potential to achieve wide coverage and sustainability but require significant partnerships between major stakeholders [93]. Local customization of interventions has been shown to be an important factor for strong community engagement [30]. Community-based interventions are not simple to design and implement, and time is required to establish robust and trusting intersectoral partnerships. Yet, as Raju (2003) cautioned, if community participation is viewed as a means to shift responsibility and costs from government to residents without providing adequate services to support residents, the likelihood of sustainability is very small [32].

As health education is a fundamental component, particularly in settings where literacy levels may be lower, careful consideration must be given to the educational approach and materials. These principles are not new in health education research [94] and underscore the need to engage with health education experts. Active engagement, ownership, and understanding of those materials by the community are important factors to take into consideration, and the diverse stakeholders each have a role to ensure those materials are adequate.

## Conclusions

Higher quality research and standard reporting of interventions are necessary if we are to successfully control in VBDs. The findings from this review included recommendations for devoting longer times to follow-up, combining human and entomological indicators in evaluating interventions, conducting more qualitative research, and using standardized tools to report intervention methods. More implementation research is needed to better understand what vector control interventions work in which contexts and, importantly, why and how. Interventions involving horizontal approaches, community participation, and social mobilization show potential, all precautions kept due to potential bias and limitations of the present review, and require sustained intersectoral collaborations between government sectors and communities to be successful.

## Box 1 priority needs for future research

- Conduct research to include sanitation and waste management.
- Include more systematically indicators of human morbidity, acceptability, sustainability and implementation indicators in intervention evaluations.
- Develop qualitative research and implementation research.
- Conduct research on ecological sustainable measures to control vector populations.
- Use and adopt high quality standards for reporting of interventions.
- Generate theory-based interventions and tools.

## Box 2 implications for public health policy and/or practice

- Promote sanitation improvements as an integrated disease management strategy.
- Redirect vertical programs towards community-based programs and intersectoral partnership.
- Consider the time and resources required to successfully implement complex interventions.
- Ensure ownership and understanding of educational materials by the community and implement interventions based on relevant health education theory.
- Reduce dependence on chemical insecticides to curb growing resistance to insecticides by adopting an eco-health perspective.
- In the context of low resource settings, may need to prioritize the most vulnerable populations.

## Additional files

Additional file 1: Multilingual abstracts in the five official working languages of the United Nations. (PDF 872 kb)

Additional file 2: Complete search strategy. (DOCX 35 kb)

Additional file 3: Data extraction grid. (XLSX 157 kb)

## Abbreviations

ASTAIRE: Analysis of the transferability and support to adaptation of health promotion interventions
LMICs: Low- and middle-income countries
MMAT: Mixed Method Appraisal Tool
TIDiER: Template for Intervention Description and Replication
VBDs: Vector-borne diseases
VERDAS: VEctor boRne DiseAses Scoping reviews
WHO: World Health Organization
