# Supplementary material for: Interventions for vector-borne diseases focused on housing and hygiene in urban areas: a scoping review
Source: Infect Dis Poverty. 2018 Sep 3;7:96. doi: 10.1186/s40249-018-0477-5 (PMC6120073; doi:10.1186/s40249-018-0477-5)
Supplement: Supplementary file 1 — Multilingual abstracts in the five official working languages of the United Nations. (PDF 872 kb) [file 40249_2018_477_MOESM1_ESM.pdf]

Translation of the abstract into the five official working languages of the United Nations

ركزت التدخلات الخاصة بالأمراض المنقولة بالنواقل على الإسكان والنظافة في المناطق الحضرية: مراجعة استطلاعية

ستيفاني ديجروتا وكايت زينسير وفاليري ريدا

نبذة مختصرة
المعلومات الأساسية: أكثر من نصف سكان العالم البشري معرضون حاليًا لخطر الأمراض المنقولة بالنواقل (VBDs)، ويتحمل أفقر الناس والمجتمعات والبلدان في العالم العبء الأكبر.
كان الهدف من هذه الدراسة هو إجراء مراجعة على تدخلات VBD ذات الصلة بالإسكان والنظافة (بما في ذلك الصرف الصحي وإدارة النفايات) في المناطق الحضرية.
الجزء الرئيسي: أجرينا مراجعة استطلاعية ، شملت بشكل منهجي البحث في المنشورات غير الرسمية التي تم استعراضها بين عامي 2000 و 2016 باستخدام خمسة قواعد بيانات علمية وقاعدة بيانات واحدة للمنشورات غير الرسمية. واستخدمت أدوات استخراج البيانات المختلفة لتشفير البيانات واستخراجها. قمنا بتقييم جودة كل دراسة باستخدام أداة تقييم الطرق المختلطة واستخلاص صفات وصفية وبيانات عن عملية التنفيذ وقابلية النقل من جميع الدراسات باستخدام قالب لتوصيف التكرار والنسخ و ASTAIRE (أداة لتحليل إمكانية نقل تدخلات تعزيز الصحة) . راجعنا 44 دراسة. وعموما ، تم الحكم على الدراسات كونها عالية الخطورة للتحيز. تشير النتائج التي توصلنا إليها إلى أن التدخّلات المتعددة الأوجه ، لاسيما التدخّلات المجتمعية ، تنطوي على إمكانات لتحقيق تأثيرات أوسع وأكثر استدامة مقارنةً بنموذج البرامج الرأسية الأحادية المكون. تميل تقييمات التدخلات متعددة الأوجه إلى تضمين التقييمات المتكاملة ، ليس فقط باستخدام مؤشرات الحشرات ، ولكن كذلك مؤشرات القبول والاستدامة.
الاستنتاجات: أبرزت هذه المراجعة الحاجة الماسة للبحوث ذات الجودة العالية في VBDs وتقارير محسنة وموحدة للتدخلات. تم العثور على ثغرات بحثية كبيرة فيما يتعلق بالبحوث النوعية وبحوث التنفيذ ، وسلطت النتائج الضوء على الحاجة إلى المزيد من التدخلات التي تركز على ممارسات الصرف الصحي والنظافة.

Translated from English version into Arabic by Rehab Rayan, proofread by Shefa'a Qudah, through

TRANSLATORS
WITHOUT BORDERS

专注于城市地区住房和卫生的媒传疾病干预措施：堪域综述

Stéphanie Degroote, Kate Zinszer and Valery Ridde

摘要
引言：目前，世界上一半以上人口正面临罹患媒传疾病（VBDs）的风险，最贫穷的人群、社区和国家的疾病负担最重。本研究旨在对城市地区住房和卫生（包括环境卫生和废弃物管理）相关的 VBD 干预措施进行综述。
主要内容：我们在 5 个科学数据库和 1 个灰色文献数据库进行了系统地检索，检索了 2000–2016 年发表的经同行评议的文献和灰色文献。使用不同的数据提取工具进行数据编码和提取。我们使用混合方法评估工具（MMAT）评估每项研究的质量，并采用干预描述和复制模板（TIDieR）

和 ASTAIRE（一个分析健康促进干预措施可转移性的工具）提取所有研究中关于实施过程和可转移性的描述性特征和数据。
共分析了 44 项研究。总体而言，这些研究被认为具有高偏倚风险。本研究结果表明，与标准的垂直单一干预措施相比，联合使用多种干预措施，尤其是基于社区的干预措施，有可能实现更广泛和更持久的影响。一般采用综合评估来评价联合使用多种干预措施的效果，包括昆虫学指标、可接受性和可持续性指标。
结论：本文强调了对 VBDs 更高质量研究，以及对干预措施的改进和标准化报告的需求。结果表明，定性研究和实施研究方面存在显著的研究差距，同时强调更多的干预措施应着眼于卫生和卫生实践方面。

Translated from English version into Chinese by Fan Yang, edited by Pin Yang

CHINESE CENTER FOR DISEASE CONTROL AND PREVENTION
NATIONAL INSTITUTE OF PARASITIC DISEASES

Interventions pour les maladies à transmission vectorielle axées sur le logement et l'hygiène dans les zones urbaines : examen de portée

Stéphanie Degroote, Kate Zinszer et Valery Ridde

Résumé
Contexte : plus de la moitié des populations humaines du monde sont actuellement exposées à des maladies à transmission vectorielle (MTV), et le fardeau le plus lourd est supporté par les populations, les communautés et les pays les plus pauvres du monde. Le but de cette étude était d'examiner les interventions MTV en matière de logement et d'hygiène (y compris l'assainissement et la gestion des déchets) dans les zones urbaines.
Structure principale: nous avons effectué un examen de la portée, qui comprenait une recherche systématique dans la littérature grise publiée entre 2000 et 2016 à l'aide de cinq bases de données scientifiques et d'une base de données pour la littérature grise. Différents outils ont été utilisés pour le codage et l'extraction des données. Nous avons évalué la qualité de chaque étude à l'aide de l'outil d'évaluation des méthodes mixtes. Nous avons également extrait des caractéristiques descriptives et des données sur le processus de mise en œuvre et la transférabilité de toutes les études à l'aide des outils Template for Intervention Description and Replication et ASTAIRE (un outil d'analyse de la transférabilité des interventions de promotion de la santé). Nous avons examiné 44 études. Dans l'ensemble, les études ont été considérées comme présentant un risque élevé de partialité. Nos résultats suggèrent que les interventions à volets multiples, en particulier les interventions communautaires, ont le potentiel d'obtenir des effets plus larges et plus soutenus que les programmes verticaux standard à composante unique. Les évaluations des interventions à volets multiples ont tendance à inclure des évaluations intégrées, utilisant non seulement des indicateurs entomologiques, mais aussi des indicateurs d'acceptabilité et de durabilité.
Conclusions: cette étude a souligné la nécessité d'améliorer la qualité de la recherche sur les MTV et d'améliorer et de normaliser les rapports sur les interventions. D'importantes lacunes en matière de recherche ont été constatées en ce qui concerne la recherche qualitative et la recherche sur la mise en

œuvre, et les résultats ont mis en évidence la nécessité de concentrer davantage les interventions sur les pratiques d'assainissement et d'hygiène.

Translated from English version into French by Louis Gauvreau, proofread by Anne Marie, through

Меры по оперативному исправлению ситуации с трансмиссивными заболеваниями, нацеленные на жилищные условия и гигиену в городских районах: аналитический обзор.

Стефани Дегрот, Кейт Зинсзер и Валери Ридд

Аннотация

Краткое описание: Больше половины населения мира в настоящее время подвергается риску заражения трансмиссивными болезнями (ТМБ), и самое тяжёлое бремя несут наиболее бедные люди, сообщества и страны в мире. Целью этого исследования было проведение анализа оперативных мер по предотвращению ТМБ, имеющих отношение к жилищным условиям и гигиене (включая санитарные условия и утилизацию отходов) в городских районах.

Основная часть: Мы выполнили аналитический обзор, включающий поиск по рецензируемой и неофициальной литературе, напечатанной между 2000 и 2016 годами, используя 5 научных баз данных и одну базу данных для неофициальных изданий. Для кодирования и извлечения данных были использованы различные средства. Мы оценивали качество каждого исследования, используя смешанный метод оценки, и извлекали описательные характеристики и данные о процессе внедрения и переносимости из всех исследований при помощи шаблона описания оперативных вмешательств и воспроизведения, а также инструмента для анализа переносимости мер по внедрению санитарии и гигиены ASTAIRE.

Мы проанализировали 44 исследования. В общем и целом, исследования были оценены как имеющие высокий риск необъективности. Наши результаты предполагают, что комплексные вмешательства, особенно на местном уровне, имеют потенциал добиться более широкого и более устойчивого эффекта, чем стандартные вертикальные однокомпонентные программы. Оценки комплексных вмешательств имеют тенденцию включать в себя интегрированные оценки, используя не только энтомологические показатели, но также показатели приемлемости и устойчивости.

Выводы: Этот анализ выдвигает на первый план существенную нужду в повышении качества исследованиях ТМБ и улучшенной и стандартизованной отчетности об оперативных вмешательствах. Существенные исследовательские пробелы были найдены в качественных исследованиях и исследованиях по внедрению, и результаты подчеркивают необходимость увеличения оперативных вмешательств, нацеленных на санитарные условия и гигиенические привычки.

Translated from English version into Russian by Akise Lyov, proofread by Natalia Potashnik, through

Intervenciones para enfermedades transmitidas por vectores centradas en la vivienda y la higiene en áreas urbanas: un estudio de campo

Stéphanie Degroote, Kate Zinszer y Valery Ridde

Resumen

Contexto: Más de la mitad de la población humana mundial está en riesgo de contraer una enfermedad transmitida por vectores (ETV) y los que más peligro corren son las personas, las comunidades y los países más pobres del mundo. El objetivo de este estudio era llevar a cabo una revisión de las intervenciones para las ETV relacionadas con la vivienda y la higiene (incluyendo la limpieza y la gestión de los residuos) en las áreas urbanas.

Cuerpo principal: Realizamos un estudio de campo que implicaba la búsqueda sistemática de literatura, tanto revisada como no revisada, publicada entre 2000 y 2016, mediante la utilización de cinco bases de datos científicas y una base de datos de literatura no revisada. Se utilizaron diferentes herramientas de extracción de datos para la codificación y extracción de los mismos. Se evaluó la calidad de cada estudio utilizando la Herramienta de Valoración de Métodos Mixtos y se extrajeron las características y datos descriptivos sobre el proceso de implementación y transferibilidad de todos los estudios utilizando el Formulario para la Descripción y Réplica de Intervenciones y la herramienta ASTAIRE (una herramienta para analizar la transferibilidad de las intervenciones de promoción sanitaria).

Se revisaron 44 estudios. En general, se consideró que los estudios tenían un alto riesgo de sesgo. Los resultados obtenidos muestran que las intervenciones polifacéticas, sobre todo las intervenciones basadas en la comunidad, tienen el potencial de alcanzar efectos más amplios y continuados que los programas verticales estándar de un solo elemento. Las evaluaciones de las intervenciones polifacéticas suelen incluir evaluaciones integradas que no solo utilizan indicadores entomológicos, sino también indicadores de aceptabilidad y sostenibilidad.

Conclusiones: Esta revisión ha subrayado la importante necesidad de realizar estudios de mejor calidad sobre las ETV y de mejorar y estandarizar la comunicación de las intervenciones. Se hallaron lagunas de investigación en lo referente a la investigación cualitativa y al estudio de la implementación, y los resultados subrayaron la necesidad de llevar a cabo más intervenciones centradas en las practicas de limpieza e higiene.

Translated from English version into Spanish by Ribcamar, proofread by Maria Schön, through
