# Supplementary material for: Interventions for vector-borne diseases focused on housing and hygiene in urban areas: a scoping review
Source: Infect Dis Poverty. 2018 Sep 3;7:96. doi: 10.1186/s40249-018-0477-5 (PMC6120073; doi:10.1186/s40249-018-0477-5)
Supplement: Supplementary file 2 — Complete search strategy. (DOCX 35 kb) [file 40249_2018_477_MOESM2_ESM.docx]

**Research topic:**

Housing, hygiene, sanitation and water, waste and infrastructure management in vector borne diseases prevention in urban areas.

**______________________________________________________________________________**

**Research question:**

What is the existent evidence related to housing, hygiene, sanitation and water, waste and infrastructure management used in preventing vector borne diseases in urban areas?

**______________________________________________________________________________**

**Specific objective:**

To conduct a scoping review to identify and synthesize existent evidence and knowledge gaps on the topic of preventive measures related to housing, hygiene, sanitation and water, waste and infrastructure management of vector borne disease in urban areas.

**Key concepts:**

1. Vector borne disease
2. Urban area
3. Housing
4. Sanitation
5. Prevention

**__________________________________________________________________**

**Key words:**

**Key Concept # 1: Vector borne disease**

**Associated Keywords:** Vector-borne disease*; communicable diseases emerging; emerging infectious disease*; resurfacing infectious disease*; communicable disease*; tropical disease*; Disease Vectors*; Insect vector; arthropod vectors; neglected tropical diseases; Sandfly fever; phelebotomus fever; bilharziasis; filariasis; chikungunya; leishmaniasis; Crimean-Congo haemorrhagic fever; Lyme disease; Relapsing fever; borreliosis; Rickettsial diseases; spotted fever; Q fever; Tick-borne encephalitis; Tularaemia; Chagas disease; American trypanosomiasis; tularaemia; malaria; dengue; rift valley fever; yellow fever; zika; japanese encephalitis; lymphatic filariasis; West Nile fever; Schistosomiasis; Sleeping sickness; African trypanosomiasis; Plague; Rickettsiosis; Onchocerciasis; river blindness; Aedes; Dog disease; mosquitoes.

**Key Concept # 2: Urban area**

**Associated Keywords:** Urban*, Urban area; urban population; urban setting; urban health; city; urbanization; urban planning; urbanism; urban morphology; urban core; urban environment; urban built environment; infrastructure; towns; cities; urban sprawl; urban landscape, urban ecosystem; metropolitan; municipality; neighbourhood; town; borough; downtown; inner city; midtown; megacity; cosmopolis; metropolis; block*, slum*

**Key concept # 3: Housing**

**Associated Keywords:** Housing *; Household level; housing system; dwellings; lodging; home*; house*; housing sector; residence; housing for elderly; habitation; pad; roof; hearth; domicile; abode; place; accommodation; chalet; cottage; bungalow; flat; condominium; apartment; townhome; townhouse; farmhouse; mansion; manor; domicile, shelter*

**______________________________________________________________________________**

**Key concept # 4: Sanitation**

**Associated Keywords:** Sanitation*; Hygiene; water sanitation; sewage; sanitation facility; wastewater management; sewers; waste infrastructure; sanitation and water management; waste disposal, sanitary sewers; drainage system; water supply and sanitation; waste management; sanitation system; household water treatment; sanitation aspect; household wastewater treatment; sanitation safety planning; toilets facilities; toilets; garbage; waste; dumping ground; landfill; excrement; ordure; feces; pit latrines; dry toilets; septic tank; onsite sewage facility

**Key concept # 5: Prevention**

**Associated Keywords:** prevention; disease vector-control; preventive health services; preventive measures; preventive intervention; preventive strategy; preventive program; integrated vector management; prevention and control; preventive activities; control measures; preventative method; primary prevention; secondary prevention; tertiary prevention; targeted prevention; global prevention; vector-borne disease prevention; control; measures, prevent*

**MeSH terms identified (From PubMed database)**

| **Keyword** | **MeSH term/ MeSH terms** |
| --- | --- |
| Vector borne disease | Communicable diseases; Disease vectors |
| Communicable diseases emerging | communicable diseases emerging |
| Communicable disease | Communicable diseases |
| Sandfly fever | Sandfly fever Naples virus; phlebovirus; phlebotomus fever |
| Disease Vectors | Disease Vectors |
| arthropod vectors | Arthropod vectors |
| Bilharziasis | Schistosomiasis |
| Filariasis | filariasis |
| chikungunya | Chikungunya virus; chikungunya fever |
| Leishmaniasis | Leishmaniasis; Leishmaniasis, Visceral |
| Crimean-Congo haemorrhagic fever | Hemorrhagic Fever Virus; Crimean-Congo; Hemorrhagic Fever, Crimean |
| Lyme disease | Lyme disease |
| Relapsing fever | Relapsing fever |
| Borreliosis | Borrelia Infections |
| Rickettsial diseases | Rickettsia; Rickettsia Infections |
| spotted fever | Rickettsia Infections; Tick-Borne infections |
| Q fever | Q Fever ; Coxiella burneti |
| Tick-borne encephalitis | Encephalitis Viruses, Tick-Borne; Encephalitis, Tick-Borne; |
| Tularaemia | Tularaemia |
| Chagas disease | Chagas disease |
| American Trypanosomiasis | Trypanosomiasis, African |
| Malaria | Malaria |
| Dengue | Dengue |
| rift valley fever | Rift valley fever; Rift valley fever virus |
| yellow fever | Yellow fever |
| Zika | Zika virus infection; Zika virus |
| japanese encephalitis | Encephalitis, Japanese |
| lymphatic filariasis | Elephantiasis, Filarial |
| West Nile fever | West Nile fever |
| Schistosomiasis | Schistosomiasis |
| Sleeping sickness | Trypanosomiasis, African |
| African trypanosomiasis | Trypanosomiasis, African |
| Plague | Plague; Yersinia pestis |
| Rickettsiosis | Rickettsia; Rickettsia Infections |
| Onchocerciasis | Onchocerciasis |
| river blindness | Onchocerciasis, Ocular |
| Aedes  Dog disease | Aedes  Dog diseases |
|  |  |
| Urban | urban population |
| Urbanization | Urbanization |
| Urban area | urban population |
| urban setting | urban population |
| urban health | urban health; urban population |
| City | Cities |
| urban planning | City planning |
| urban morphology | urban population |
| urban core | urban population; urbanization |
| neighborhood | Residence characteristics |
| urban built environment  infrastructure  cities  urban sprawl  urban landscape  metropolitan  municipality  borough  downtown  inner city  block*  slum*  sanitation facility  sewage  feces | Residence characteristics  ---  cities  urbanization  city planning  urban population  ---  ---  --  Urban population  --  Urban population  toilet facilities; sanitation  sewage  feces |
| Housing | Housing; public housing |
|  |  |
| Prevention  Preventive services  Primary prevention  Secondary prevention  Tertiary prevention | Prevention and control  Preventive health services  Primary prevention  Secondary prevention  Tertiary prevention |

**MeSH terms (From Global health)**

| **Keyword** | **MeSH term/ MeSH terms** |
| --- | --- |
| Vector borne disease | Communicable diseases; Disease vectors |
| Communicable diseases emerging | Infectious diseases |
| Emerging infectious disease | Infectious diseases |
| Communicable disease | Infectious diseases |
| Sandfly fever | Sandfly fever |
| Disease Vectors | Disease Vectors |
| arthropod vectors | Arthropod vectors |
| Bilharziasis | Schistosomiasis |
| Filariasis | filariasis |
| chikungunya | Chikungunya virus |
| Leishmaniasis | Leishmaniasis |
| Crimean-Congo haemorrhagic fever | Crimean-Congo; Hemorrhagic fever virus |
| Lyme disease | Lyme disease |
| Relapsing fever | Relapsing fever |
| Borreliosis | Borrelia burgdorferi |
| Rickettsial diseases | Rickettsial disease |
| spotted fever | Spotted fever |
| Q fever | Q Fever |
| Tick-borne encephalitis | Tick-Borne encephalitis virus |
| Tularaemia | Tularaemia |
| Chagas disease | Chagas’ disease |
| American Trypanosomiasis | African Trypanosomiasis |
| Malaria | Malaria |
| Dengue | Dengue |
| rift valley fever | Rift valley fever |
| yellow fever | Yellow fever |
| Zika | Zika virus |
| japanese encephalitis | Encephalitis, Japanese |
| lymphatic filariasis | Elephantiasis, Filarial |
| West Nile fever | West Nile fever |
| Schistosomiasis | Schistosomiasis |
| Sleeping sickness | African Trypanosomiasis |
| African trypanosomiasis | African Trypanosomiasis |
| Plague | Plague |
| Rickettsiosis | Rickettsial disease |
| Onchocerciasis | Onchocerciasis |
| river blindness | Onchocerciasis |
| Aedes  Dog disease | Aedes  Dog diseases |

**MeSH terms (From EMBASE database)**

| **Keyword** | **MeSH term/ MeSH terms** |
| --- | --- |
| Vector borne disease | Communicable diseases; Disease vectors |
| Communicable diseases emerging | Infectious diseases |
| Emerging infectious disease | Infectious diseases |
| Communicable disease | Infectious diseases |
| Sandfly fever | Sandfly fever |
| Disease Vectors | Disease Vectors |
| arthropod vectors | Arthropod vectors |
| Bilharziasis | Schistosomiasis |
| Filariasis | filariasis |
| chikungunya | Chikungunya virus |
| Leishmaniasis | Leishmaniasis |
| Crimean-Congo haemorrhagic fever | Crimean-Congo; Hemorrhagic fever virus |
| Lyme disease | Lyme disease |
| Relapsing fever | Relapsing fever |
| Borreliosis | Borrelia burgdorferi |
| Rickettsial diseases | Rickettsial disease |
| spotted fever | Spotted fever |
| Q fever | Q Fever |
| Tick-borne encephalitis | Tick-Borne encephalitis virus |
| Tularaemia | Tularaemia |
| Chagas disease | Chagas’ disease |
| American Trypanosomiasis | African Trypanosomiasis |
| Malaria | Malaria |
| Dengue | Dengue |
| rift valley fever | Rift valley fever |
| yellow fever | Yellow fever |
| Zika | Zika virus |
| japanese encephalitis | Encephalitis, Japanese |
| lymphatic filariasis | Elephantiasis, Filarial |
| West Nile fever | West Nile fever |
| Schistosomiasis | Schistosomiasis |
| Sleeping sickness | African Trypanosomiasis |
| African trypanosomiasis | African Trypanosomiasis |
| Plague | Plague |
| Rickettsiosis | Rickettsial disease |
| Onchocerciasis | Onchocerciasis |
| river blindness | Onchocerciasis |
| Aedes  Dog disease | Aedes  Dog diseases |
